# DNA-intercalating antiphage molecules trigger abortive infection through mutual destruction and synergize with bacterial immunity

**DOI:** 10.1073/pnas.2602073123

**Published:** 2026-06-03

**Authors:** Larissa Ernst, Cornelia Gätgens, Bente Rackow, Nadiia Pozhydaieva, Elyès Gaaloul, Aileen Krüger, Johannes Seiffarth, Michelle Bund, Vivien Joisten-Rosenthal, Dietrich Kohlheyer, Björn Usadel, Alexander Harms, Katharina Höfer, Julia Frunzke

**Affiliations:** ^a^ Institute of Bio- und Geosciences, IBG-1: Biotechnology, Forschungszentrum Jülich, Jülich 54425, Germany; ^b^Faculty of Mathematics and Natural Sciences, Institute for Microbial Interactions, Heinrich-Heine-University Düsseldorf, Düsseldorf 40225, Germany; ^c^ Max Planck Institute for Terrestrial Microbiology, Marburg 35037, Germany; ^d^ Department of Pharmacy, Institute of Pharmaceutical Biology and Biotechnology, Philipps-Universität Marburg, Marburg 35037, Germany; ^e^Faculty of Mathematics and Natural Sciences, Institute for Biological Data Science, Heinrich-Heine-University Düsseldorf, Düsseldorf 40225, Germany; ^f^ Institute of Bio- and Geosciences, IBG-4: Bioinformatics, Forschungszentrum Jülich, 52425 Jülich, Germany; ^g^ Department of Health Sciences and Technology, Institute of Food, Nutrition, and Health, ETH Zürich, Zürich 8092, Switzerland; ^h^ Center for Synthetic Microbiology, Bacterial Epitranscriptomics, Philipps-Universität Marburg, Marburg 35037, Germany

**Keywords:** bacteriophage, bacterial immunity, phage defense, daunorubicin, T5

## Abstract

Bacteria deploy diverse antiphage defense systems, including small bioactive molecules providing protection at the multicellular level. DNA-intercalating anthracyclines, such as daunorubicin, exhibit broad antiphage activity, but the underlying mechanism has remained elusive. Here, we systematically screened the *Escherichia coli* BASEL phage collection to elucidate the mode of action of DNA-intercalating antiphage molecules. We identified taxonomically distinct clusters of susceptible viral groups and show that for the *Tequintavirus* Bas33 (*Markadamsvirinae*), daunorubicin blocks infection after first-step transfer. In the presence of daunorubicin, continued expression of pre-early genes leads to abortive infection via “mutual destruction,” where both phage and host succumb. Analogous abortive-infection phenotypes occur across taxonomically diverse phages exposed to chemically distinct DNA-intercalating molecules. Notably, we show that daunorubicin synergizes with downstream nucleic acid-targeting defenses underscoring context-dependent outcomes. Together, these findings reveal how chemical defense contributes to the multilayered antiviral immunity and highlight the intricate interplay between mechanistic inhibition and infection outcome.

## Significance

Bacteria deploy chemical defenses to protect against viral infection, yet how such small molecules shape infection outcomes has remained unclear. We show that DNA-intercalating anthracyclines interfere with early stages of phage infection, triggering abortive infection through “mutual destruction” driven by toxic phage products rather than host suicide pathways. By systematically mapping phage susceptibility and dissecting the infection process, we reveal that chemical defense via anthracyclines acts at defined stages of the phage life cycle and can synergize with canonical nucleic acid-targeting immune systems to promote host survival. These findings uncover a mechanistic basis for small-molecule antiviral immunity and highlight how layered defenses collectively determine infection fate.

The ongoing evolutionary arms race between bacteria and phages drives a dynamic process of coevolution. Bacteria have evolved a wide array of antiphage defense systems to counter viral predation, while phages, in turn, develop countermeasures to overcome these protective barriers (1–4). The investigation of bacterial antiphage defense systems has emerged as a rapidly expanding research field, not only for advancing our fundamental understanding of virus–host interactions but also for the potential biotechnological exploitation of these systems as novel molecular tools. To date, an ever-growing number of defense mechanisms have been identified, extending beyond the classic nucleic acid-targeting restriction-modification (RM) systems and CRISPR-Cas to encompass a striking diversity of strategies, including abortive infection (Abi), nucleotide depletion, and cyclic-oligonucleotide-based antiphage signaling systems (CBASS) (5).

Abi had initially been introduced as a broad term describing the phenomenological category of antiphage defense systems inducing programmed cell death (PCD) after the detection of a viral infection (6). In recent years, numerous novel antiphage systems have been discovered that appear to function as a “last line of defense” by triggering host cell death. Yet, emerging evidence suggests a more complex relationship between the underlying mechanism of defense and the resulting infection phenotype. In a recent opinion article, Aframian and Eldar argue for disentangling mechanism from phenotype. They explicitly distinguish between cell death via the classical PCD targeting host components and death of the host driven by the action of phage components even after successful inhibition of viral infection, a process they define as “mutual destruction” (7).

In addition to RNA- and protein-based defense systems, small, bioactive molecules naturally produced by *Streptomyces* spp. have been shown to act as potent antiphage agents. Reported examples include in particular DNA-intercalating molecules, such as anthracyclines, as well as diverse molecules belonging to the class of aminoglycosides (8–12). Due to their secretion into the environment, this so-called chemical defense via small molecules bears the potential to confer protection against viral infection at the community level (13, 14).

Daunorubicin (Dau), also referred to as daunomycin, was previously reported to exhibit broad antiphage activity (9). This anthracycline is a well-established chemotherapeutic agent, primarily used to treat myeloblastic and acute lymphoblastic leukemias. Its cytotoxic activity stems from its ability to inhibit human topoisomerase II through the intercalation of its planar group between adjacent DNA/RNA base pairs, thereby preventing the progression of nucleic acid replication (15). DNA intercalating agents, which perturb DNA metabolism in both eukaryotic and prokaryotic cells (15, 16), represent promising candidates in the search for novel antiphage compounds. While their antiphage activity was recognized already in the past century (10, 11), it was only recently that they have been appreciated as part of the natural *Streptomyces* antiviral immune system. The mechanism by which daunorubicin inhibits phage infection and the factors determining viral susceptibility to daunorubicin - even at concentrations without significant effects on the host - have remained elusive. Previous work has shown that daunorubicin acts at an early stage of the phage replication cycle after DNA injection but prior to the onset of genome replication (9).

To unravel the mode of action of daunorubicin and to identify phage determinants conferring sensitivity to DNA-intercalating antiphage molecules, we harnessed the *Escherichia coli* BASEL (Bacteriophage Selection for your Laboratory) collection (17). Systematic screenings revealed taxonomically related clusters of phage families showing high susceptibility to the antiphage action of daunorubicin. Exemplified for tequintaviruses (*Demerecviridae*/ *Markadamsvirinae*), we further demonstrate that antiphage activity is mediated through mutual destruction. Daunorubicin caused the blockage of phage infection after first-step transfer (FST) at the level of pre-early gene expression, which ultimately imposes a lethal outcome on the bacterial host. We further demonstrate that the defense phenotype is context dependent, as daunorubicin synergizes with downstream nucleic acid-targeting systems. These results emphasize the intricate interaction between different lines of defense ultimately defining bacterial antiviral immunity.

## Results

### Taxonomically Distinct Pattern of Daunorubicin Sensitivity among Phage Families.

Previous studies have identified the DNA-intercalating compound daunorubicin as a broad-spectrum antiphage agent that blocks an early step of infection (9). To elucidate its mode of action and uncover phage-encoded determinants of sensitivity, we leveraged the *E. coli* BASEL phage collection, which enables systematic analyses across a wide taxonomic diversity of phages (17). As bacterial host, we utilized the *E. coli* K-12 MG1655 ΔRM strain lacking all native restriction-modification systems (ΔRM), the abortive infection systems PifA and RexAB as well as the O-antigen glycan barrier. This strain provides a genetically simplified background, minimizing confounding interactions with native bacterial immunity and allowing for a more direct assessment of daunorubicin’s antiphage activity (17).

**Fig. 1.**
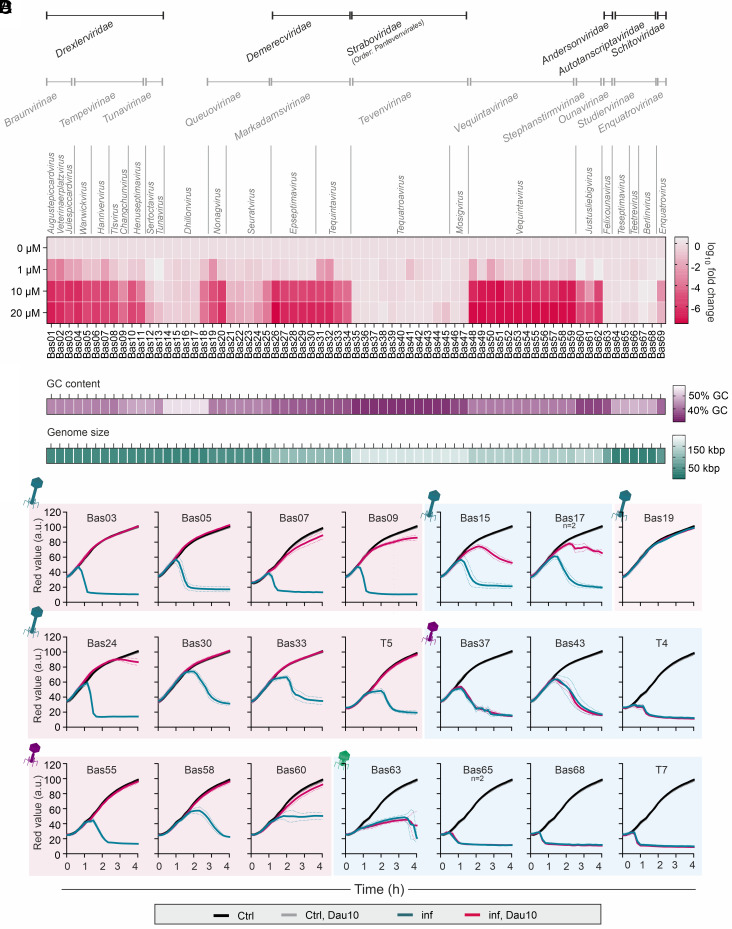
Screening of the BASEL phage collection revealed taxonomically distinct patterns of phages with high sensitivity to daunorubicin. (*A*) Heat map showing the mean log_10_ FC in EOP upon addition of increasing daunorubicin concentrations to LB double-agar overlay assays inoculated with in *E. coli* K-12 MG1655 ΔRM (n = 2, mean values shown). (*B*) GC-content and (*C*) genome size of the different BASEL phages [Maffei et al. (17)]. (*D*) Liquid infection experiments of selected phages as representatives of the different taxonomic groups. Infection was performed at an MOI of 0.1 in LB medium with and without 10 µM daunorubicin (n = 3, mean values with SD shown). Morphotypes of the different phage families are indicated with respective icons (blue: siphovirus, purple: myovirus, green: podovirus). Phages showing sensitivities toward daunorubicin in the plate screening are shaded red, whereas resistant phages are shaded blue.

Prior to the screening, we tested the impact of daunorubicin on growth of *E. coli* K-12 MG1655 ΔRM and found only a minor influence at concentrations above 100 µM (MIC > 150 µM). For the screening of the BASEL collection, we therefore applied significantly lower concentrations. Exposure of the phage collection to increasing concentrations (0 to 20 µM) of daunorubicin during infection of *E. coli* K-12 MG1655 ΔRM revealed distinct sensitivity patterns (Fig. 1*A*). Notably, members of the tested genera within the *Drexlerviridae* phage family exhibited markedly reduced efficiency of plating (EOP) at 10 µM daunorubicin. Similar sensitivity was observed for tested members of the *Demerecviridae* family and the *Vequintavirinae* subfamily. In contrast, an intermediate level of susceptibility was noted for the *Seuratvirus* genus within the *Queuovirinae* subfamily. Conversely, phages belonging to the *Dhillonvirus* genus, the two genera of the *Tevenvirinae* subfamily, and the three tested genera of the *Studiervirinae* subfamily displayed pronounced resistance to daunorubicin in this host genetic background. Notably, neither GC content nor phage genome size showed significant correlation with the observed daunorubicin sensitivity patterns (Fig. 1 *B* and *C*).

These findings were further validated using infection assays in liquid cultures at a multiplicity of infection (MOI) of 0.1 in the presence of 10 µM daunorubicin (Fig. 1*D*). Except for Bas19, which caused no growth defect upon phage addition even in the absence of daunorubicin, infection dynamics were largely consistent with the results obtained from the plate screenings. Phages classified as daunorubicin-sensitive caused reduced or even no detectable host cell lysis under drug exposure, while tested phages belonging to genera within the resistant *Tevenvirinae* and *Studiervirinae* displayed no significant alteration in infection efficiency. Interestingly, although *Dhillonvirus* phages Bas15 and Bas17 were classified as resistant, they exhibited a mild yet reproducible reduction in host lysis in liquid infection assays, indicating partial susceptibility to daunorubicin without complete inhibition of phage propagation.

### High Resistance of *Tevenvirinae* Phages Is Not Conferred through Genome Hypermodification.

Apart from genome size and GC content, covalent base modifications could be another factor influencing the antiviral activity of DNA intercalating compounds. The daunorubicin-resistant *Tevenvirinae*, including the prominent *E. coli* model phage T4, are known for their large genome size of >160 kbp and their high resistance to DNA-targeting immune mechanisms such as restriction-modification systems. This resistance is attributed to the hypermodification of cytosines by hydroxymethyl glycosylation for the *Tequatrovirus* genus and hydroxy arabinosylation for the *Mosigvirus* genus (18–20). To test whether these base modifications affect infectivity in the presence of daunorubicin, we compared the infection dynamics of T4 and T4 Δα-/β-gt (Δgt), which lacks the α-/β-glycosyltransferases responsible for the final glycosylation of hydroxylmethyl-dCTPs (provided by Marianne De Paepe) (*SI Appendix*, Fig. S1*A*). In the presence of 20 µM daunorubicin, no difference in infectivity was observed between the tested conditions for either T4 variant (*SI Appendix*, Fig. S1 *B* and *C*) on *E. coli* K-12 MG1655 ΔRM. Based on these results, we concluded that the DNA modification via hydroxymethyl glycosylation does not severely impact the phage susceptibility to daunorubicin.

### Daunorubicin Causes an Abortive Infection Phenotype.

**Fig. 2.**
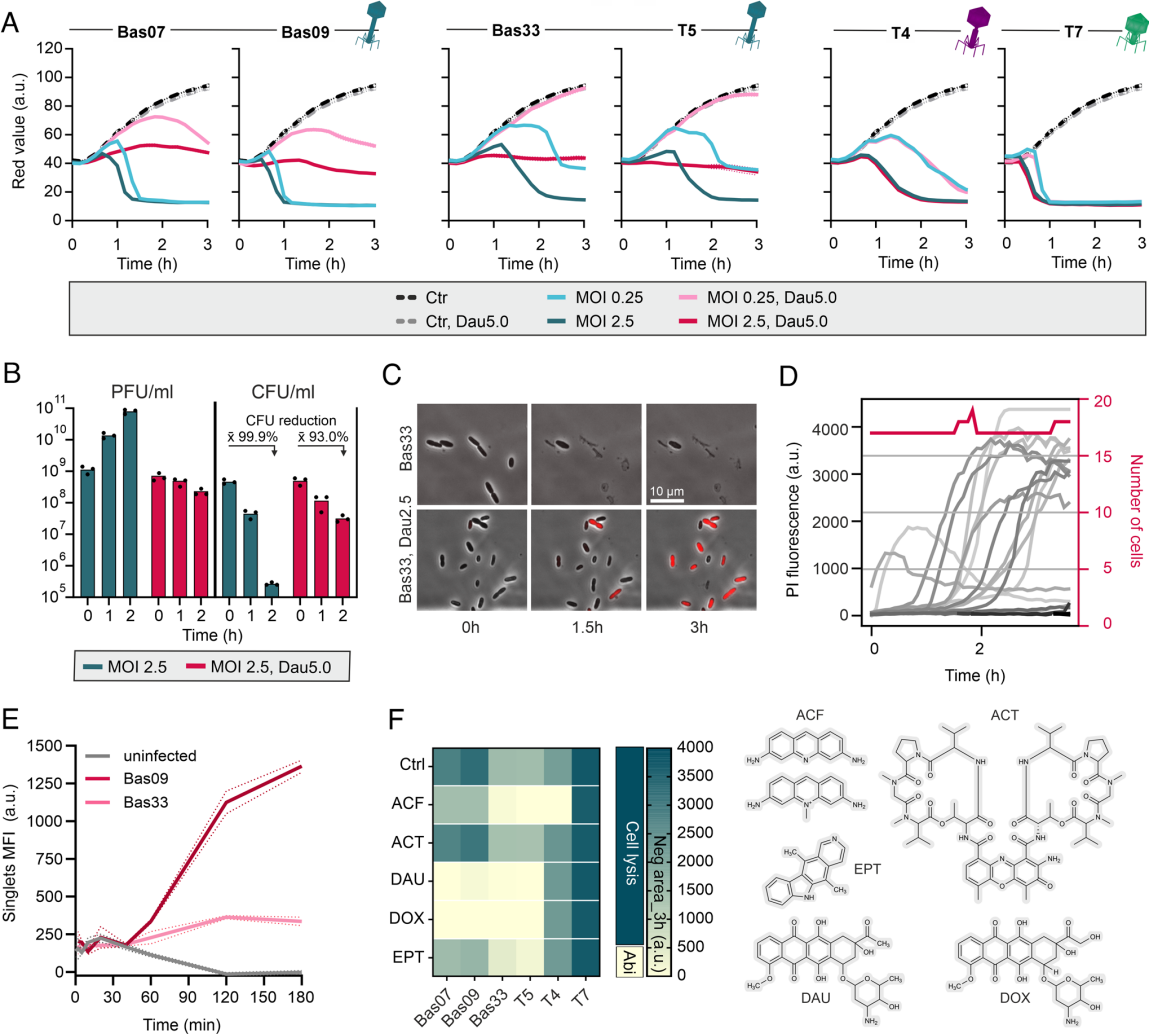
Infection in the presence of daunorubicin causes an abortive infection-like phenotype. (*A*) Liquid infection assays of selected phages in *E. coli* K-12 MG1655 ΔRM in the presence and absence of 5 µM daunorubicin at low and high MOIs of 0.25 and 2.5, respectively (n = 3, mean values with SD shown). (*B*) PFU and CFU counts upon infection with Bas33 ± 5 µM daunorubicin. The mean values (x̄) of the percentage reduction in CFU/mL are shown. CFU counts were determined by spotting a decimal dilution series on LB agar plates containing 0.1% citrate for phage inactivation. (*C*) Single-cell analysis of *E. coli* K-12 MG1655 ΔRM infected with Bas33 (*Tequintavirus*) in microfluidic chips ± 2.5 µM daunorubicin. Complete time lapse videos are provided in Movies S1 and S2. Propidium iodide staining (red fluorescence) was used to visualize increased membrane permeability and cell death. The same LUT settings were applied to all images. (*D*) PI fluorescence and number of cells upon infection of *E. coli* K-12 MG1655 ΔRM with Bas33 in the presence of 2.5 µM daunorubicin in microfluidic chips, shown for one representative single replicate. (*E*) Uptake of daunorubicin by *E. coli* K-12 MG1655 ΔRM upon phage infection at an MOI of 2.5 in the presence of 5 µM daunorubicin, measured as cell fluorescence via flow cytometry (488 nm laser) (n = 3, mean values with SD shown). (*F*) Heat map showing the negative area (a.u.) upon infection with selected Basel phages in the presence of different DNA-intercalating compounds (ACF: acriflavine, 5 µg/mL; ACT: actinomycin D; 5 µM; DAU: daunorubicin, 5 µM; DOX: doxorubicin, 5 µM; EPT: elipticine, 5 µM). Negative areas describe the mean area between the baseline at t_0_ and the growth curves as schematically indicated in *SI Appendix*, Fig. S3 and are categorized as follows: Negative Areas of ≤500 indicate an Abi-like phenotype, while increasing negative areas >500 indicate phage-induced cell lysis as for the compound-free control (ctrl) (n = 3). Structures of the respective intercalators are indicated.

Daunorubicin efficiently inhibits phage infection of all genera of phages tested within the *Drexlerviridae* or *Demerecviridae* (Fig. 1). To gain insights into the defense phenotype, infection was performed at low and high MOIs of 0.25 and 2.5, respectively (Fig. 2*A*). In the case of the sensitive phages Bas07 and Bas09 of the *Tempevirinae* subfamily (of *Drexlerviridae* family), as well as Bas33 and T5 of the *Markadamsvirinae* subfamily (of *Demerecviridae* family), infection at a low MOI of 0.25 resulted in a delayed or even absent culture collapse after addition of daunorubicin (light red vs. light blue line). Interestingly, infection in the presence of daunorubicin at an MOI of 2.5 transferred cells into a growth-arrested state (pink vs. dark blue line), which is indicative for an abortive infection phenotype. In the absence of daunorubicin, infection led to rapid culture clearance. In contrast, resistant phages such as T4 from the *Tevenvirinae* subfamily (family *Straboviridae*) and T7 from the *Studiervirinae* subfamily exhibited an identical infection dynamics, regardless of the presence of daunorubicin at either MOI.

Focusing on Bas33, growth patterns aligned with plaque- (PFU) and colony-forming units (CFU) counts. While CFU declined with rising PFUs under normal infection conditions due to cell lysis and progeny release, no phage amplification occurred with daunorubicin, though CFUs still dropped by ~93% after 120 min (Fig. 2*B*). Time-resolved single-cell microscopy confirmed the growth stagnation, showing further an increased membrane permeability and subsequent cell death in the presence of daunorubicin, as indicated by the applied propidium iodide staining and the increased daunorubicin uptake at later stages of infection (Fig. 2 *C*–*E* and *SI Appendix*, Fig. S2).

In addition to analyzing the effect of daunorubicin on infection dynamics, we also examined the effect of other DNA-intercalating agents, including doxorubicin (anthracycline), acriflavine (acridine derivative), actinomycin D (polypeptide antibiotic), and ellipticine (tetracyclic alkaloid). Negative areas, defined as deviations of the growth curves below the baseline during a 180 min infection period, were quantified and classified as ≤500 (a.u.), indicative for an Abi-like phenotype, or >500 (a.u.), indicative for cell lysis (Fig. 2*F* and *SI Appendix*, Fig. S3). As expected, doxorubicin showed almost identical effects on phage infection as daunorubicin. Interestingly, ellipticine and acriflavine exhibited no phage-inhibiting activity against the *Drexlerviridae* phage Bas07 and Bas09, but against the *Demerecviridae* phages Bas33 and T5. As expected, despite resistance to all other tested compounds, infection of phage T4 was inhibited by acriflavine, which can be traced back to the presence of the *ac* gene in the T4 genome. It is assumed that this gene interacts with the AcrAB-TolC efflux pump within the bacterial membrane, blocking acriflavine export and thus impacting DNA replication by elevating intracellular acriflavine levels (21, 22). Contrary to this, actinomycin D showed no activity against all tested phages and T7 showed no susceptibility to all tested intercalators.

Previous work on phage λ showed full restoration of bacterial growth with 40 µM daunorubicin at MOI 10 (9). At low MOIs of 0.25, we confirmed inhibition by 5 µM daunorubicin, eliminating phage-induced growth defects. However, at MOI 2.5, cells entered growth arrest after a brief increase in density (~150 min) in *E. coli* K-12 MG1655 ΔRM and BW25113 (*SI Appendix*, Fig. S4).

### Daunorubicin Blocks Infection of the *Tequintavirus* Bas33 after FST Causing Mutual Destruction via Expression of Pre-Early Genes.

**Fig. 3.**
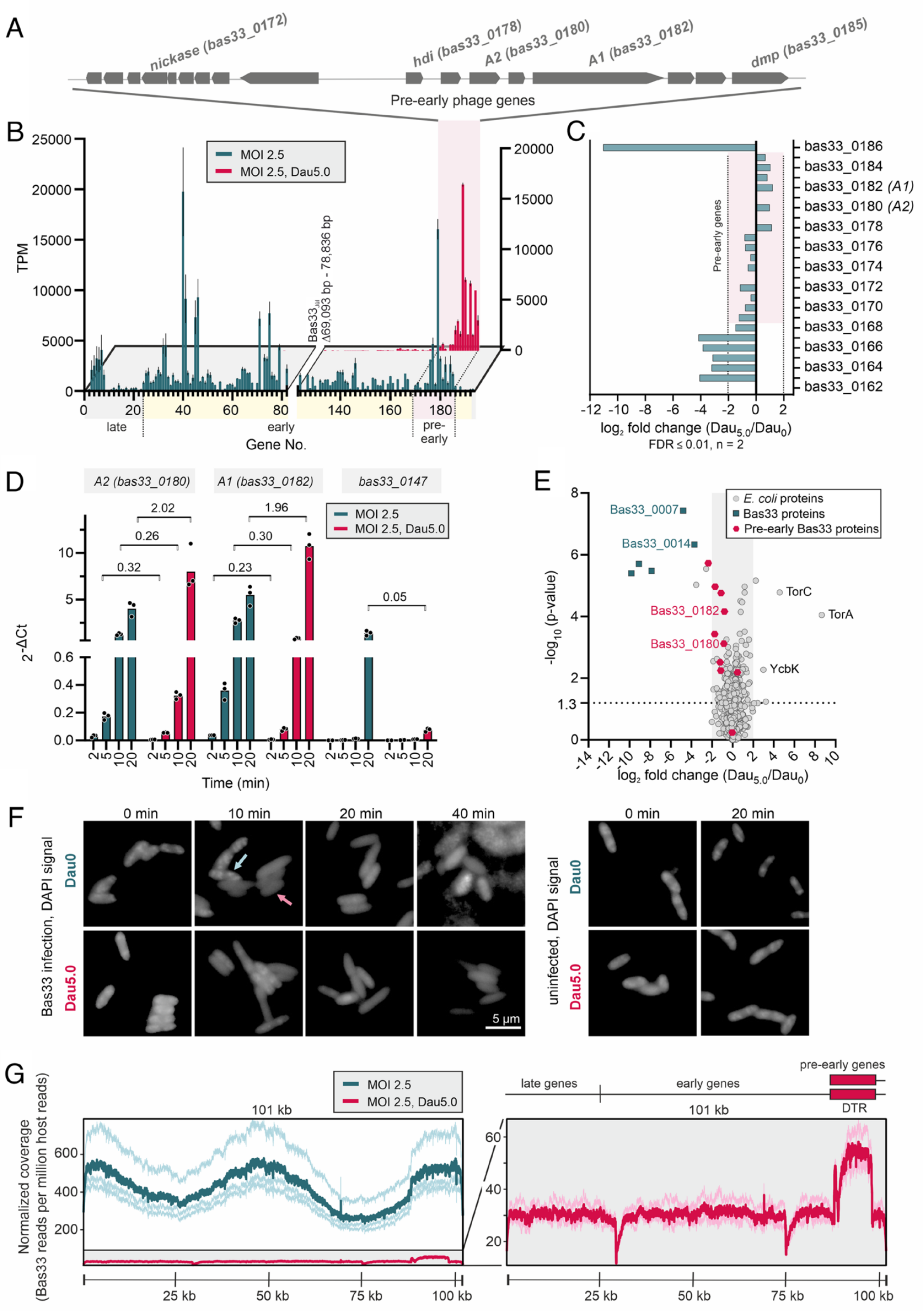
Transcriptomics and proteomics revealed a blockage of Bas33 phage gene expression after FST. (*A*) Pre-early gene region (8%) of the Bas33 genome (*Tequintavirus*) with annotation of genes of known function. (*B*) Mean values of detected phage transcripts (TPM) after 20 min of Bas33 infection using the *E. coli* K-12 MG1655 ΔRM as host background. Predicted categorization into pre-early, early, and late phage genes is highlighted alongside the gene numbers (n = 2, mean values with SD shown). (*C*) Zoom-in into log_2_ FC (Dau_5_/Dau_0_) of the pre-early gene region within the Bas33 genome showing no significant changes in transcription upon addition of daunorubicin. (*D*) RT-qPCR showing time-resolved transcription of selected pre-early phage genes (Bas33_0180, coding for A2 protein, and Bas33_0182, coding for A1 protein) in comparison to an early phage gene (Bas33_0147, putative homing endonuclease) in the presence and absence of 5 µM daunorubicin. The relative copy number (GOI to housekeeping gene *atpD* of *E. coli* K-12 MG1655 ΔRM) is shown as 2^−ΔCt^ for three independent biological replicates measured as technical duplicates. Mean values of calculated FC (Dau_5_/Dau_0_) at the different time points are indicated above. (*E*) Volcano plot showing the proteome profile as log_2_ FC in protein abundance (Dau_5_/Dau_0_). Thresholds were set to log_2_ FC of 2 and −log_10_ (*p*-value) of 1.3 (moderated *t* test, n = 3). Only proteins with an average peptide count of at least two across replicates were considered reliably detected and are included in this plot. (*F*) Fluorescence microscopy showing DAPI-stained *E. coli* K-12 MG1655 ΔRM cells at different stages of Bas33 infection in the presence and absence of 5 µM daunorubicin in comparison to uninfected controls. (*G*) Normalized genome coverage after Oxford Nanopore sequencing of intracellular and cell-associated phage DNA of Bas33-infected *E. coli* K-12 MG1655 ΔRM (n = 3), expressed as reads per million host-mapped reads. Predicted DTRs covering the pre-early phage gene region are indicated. Thin lines represent three individual replicates, while bold lines indicate the mean normalized coverage per condition.

To gain deeper mechanistic insights, we determined transcriptomic and proteomic changes in the intermediate stages of bacteriophage infection with and without daunorubicin treatment to obtain an initial snapshot of the genome-wide phage transcription profile. We focused on the daunorubicin-sensitive *Tequintavirus* Bas33, known to infect via a two-step delivery of the phage genome (23). RNA sequencing (RNA-seq) revealed distinct differences in the transcription profile of phage genes after 20 min of infection in the presence and absence of daunorubicin. While late genes coding for structural proteins, such as *bas33_0007* (major capsid protein) or *bas33_0014* (tail tube protein), were already actively transcribed under normal infection conditions, the presence of daunorubicin revealed a sharply defined expression pattern, with transcription largely restricted to the pre-early phage genes (Fig. 3 *A* and *B*). These genes are located in the first 9% of the phage genome, which is injected during the FST. Of these 17 pre-early gene products, A1 of the related model phage T5 was shown to be essential for host DNA degradation, while both A1 and A2 are essential for the second-step transfer (SST). The deoxyribonucleotide 5′-monophosphatase (Dmp) promotes infection by facilitating further degradation of host-derived nucleotides during host takeover leading to the release of free bases into the extracellular space (23). Zooming in into the log_2_ FC (Dau_5_/Dau_0_; FDR ≤ 0.01) of the pre-early phage region confirmed the comparable gene expression between both conditions, showing log_2_ FC below a threshold of ±2 (Fig. 3*C*). Time-resolved analysis of pre-early gene transcription via RT-qPCR revealed further that addition of daunorubicin led to an up to 4.5-fold reduction in transcripts of *A1 (bas33_0182)* and *A2 (bas33_0180)* within the first 10 min of transcription, while respective transcript levels exceeded the ones of the control conditions at 20 min post infection. In contrast, early gene expression *(bas33_0147)* was only detected after 20 min, with transcripts levels being 20-fold higher in the absence of daunorubicin (Fig. 3*D*). Based on the measured Ct values, transcript levels of the *E. coli* housekeeping gene *atpD* remained stable throughout the 20 min time course under the applied conditions, supporting its use as a reference gene in RT-qPCR, despite the expected degradation of *E. coli* DNA.

Remarkably, the host transcriptome of the *E. coli* K-12 MG1655 ΔRM strain exposed less differences with only <20 genes being markedly upregulated (log_2_ FC ≥ 2, FDR ≤ 0.01) and 50 genes being significantly downregulated (log_2_ FC ≤ −2, FDR ≤ 0.01; Dataset S1). Among the upregulated genes, the highest log_2_ FC (>4.0) was detected for parts of the anaerobic respiratory chain (*torAC*) and different transposase genes as well as the putative fimbrial protein-encoding gene *ybgD*. The observed transcriptional pattern was consistent with the proteomic data, showing a similar protein abundance for the pre-early phage gene products. Without daunorubicin treatment, we reliably detected 122 Bas33 phage proteins. By contrast, in the presence of daunorubicin, only 15 phage proteins were reproducibly detected: Ten of these corresponded to pre-early gene products (Fig. 3*E*), while three were structural proteins potentially deriving from adsorbed phage particles, in addition to one putative DNA-binding protein and one hypothetical protein.

Toxic effects of pre-early phage proteins such as A1, mediated through host DNA degradation, were assessed by DAPI staining over the course of infection. At early time points, distinct DAPI foci corresponding to chromatin structures were observed. These signals progressively became more diffuse under both infection conditions, indicating ongoing host DNA degradation. After 40 min of infection, control cells exhibited lysis, whereas daunorubicin-treated cells displayed a diffuse DAPI signal without complete cellular disintegration (Fig. 3*F*).

Furthermore, we examined phage DNA replication using long-read sequencing (Fig. 3*G*). When reads were mapped to the Bas33 genome, coverage was markedly higher under normal infection conditions, consistent with ongoing bidirectional replication. In contrast, in the presence of daunorubicin, coverage remained low and largely uniform across the genome, indicating that replication does not proceed. Notably, the pre-early phage gene region showed approximately twofold higher coverage relative to the rest of the genome, with sharp coverage drops at both boundaries. This pattern is readily explained by the presence of two copies of this region in the Bas33 genome, corresponding to the direct terminal repeat (DTR) for genome circularization, as described for the model phage T5 (23, 24). Additionally, the two distinct drops in the coverage plot may reflect single-stranded DNA (ssDNA) nicks, which are introduced into the 3′-5′ strand of T5 genomes prior to encapsidation (23). Overall, the sequenced DNA under drug exposure likely represents a composite signal comprising phage DNA that has been injected but not replicated, as well as DNA originating from adsorbed and/or stalled phage particles following FST. Consequently, no definitive conclusion can be drawn regarding the extent or completion of DNA injection under daunorubicin treatment.

### Synergy between Daunorubicin-Mediated Defense and Nucleic Acid–Targeting Defense Systems.

**Fig. 4.**
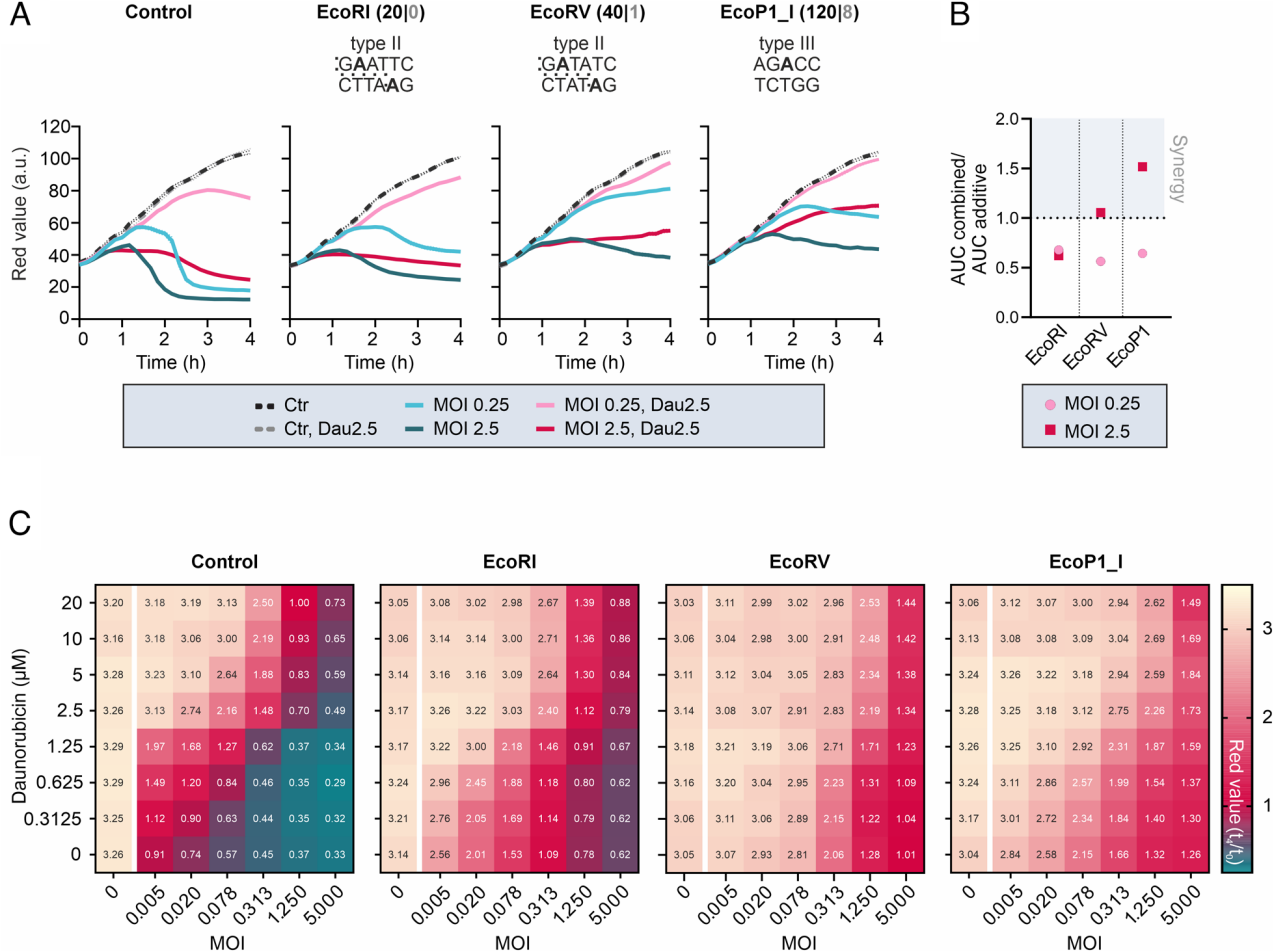
DNA-targeting defense systems show synergistic effects with daunorubicin. (*A*) Liquid infection assays of the phage Bas29 infecting *E. coli* K-12 MG1655 ΔRM strains carrying different RM systems (control = empty plasmid). Assays were performed in the presence and absence of 2.5 µM daunorubicin at low and high MOIs of 0.25 and 2.5 (n = 3, mean values with SD shown). Numbers of recognition sites of the respective RM systems in the entire genome and the pre-early gene region are indicated in black and gray, respectively. (*B*) Calculation of synergy between daunorubicin and the respective RM systems based on the “Area under the curve (AUC)” according to Wu et al. (27) using ‘Red values’ recorded at t_0_ as baseline. Ratios were calculated from AUC mean values (n = 3). (*C*) Checkerboard-like assays combining different MOIs of the phage Bas29 and different daunorubicin concentrations in the respective *E. coli* strains with distinct RM antiphage defense backgrounds. The panel shows the FC in ‘Red values’ after 4 h of cultivation and infection, with the calculated values indicated in the heatmaps.

Since DNA-intercalating compounds block phage infection at an early stage, we hypothesized that they could act synergistically with downstream DNA-targeting defense systems. Such systems have also been shown to primarily attack the pre-early phage gene region of tequintaviruses (25, 26). To test this hypothesis, we selected a representative set of RM systems and evaluated their activity in combination with daunorubicin in a defined strain background. To this end, respective systems - showing either no, intermediate, or strong defense against selected phages (17) - were expressed from a low-copy plasmid under the native promoter in the *E. coli* K-12 MG1655 ΔRM strain, and infections were performed at low and high MOIs by initially focusing on *Markadamsvirinae* subfamily within the *Demerecviridae*. However, members of the *Tequintavirus* genus, including phage Bas33, are largely resistant to RM systems, therefore we employed phage Bas29, a representative of the *Epseptimavirus* genus featuring sensitivity to RM type II system EcoRV, and intermediate sensitivity to the type II and type III RM systems EcoRI and EcoP1_I, respectively (17). As expected, due to the absence of recognition sites within the pre-early phage region, expression of EcoRI showed only minor difference compared to the growth control and retained the Abi-like phenotype at high MOIs and 2.5 µM daunorubicin. An intermediate effect was observed for the type II RM system EcoRV, preventing indeed culture collapse in the absence of daunorubicin for low MOIs, but showing no strong effect on both conditions at high MOIs. The most pronounced effect was observed for EcoP1_I, preventing the growth arrest at high MOIs in the presence of daunorubicin via synergistic interaction with daunorubicin as checked according to Wu et al. (27) (Fig. 4 *A* and *B*). This is assumed to be conferred through the presence of eight recognition sites already in the pre-early phage gene region.

To assess the synergistic effects more broadly, checkerboard-like assays combining different MOIs (0.005 to 5.0) with daunorubicin concentrations ranging from 0.3 to 20 µM were performed and FC in ‘Red values’ after 4 h was calculated to assess infection outcomes (Fig. 4*C*). Control assays with the *E. coli* K-12 MG1655 ΔRM strain demonstrated that high MOIs combined with low daunorubicin concentrations promoted phage-induced cell lysis, whereas low MOIs and elevated daunorubicin concentrations favored host survival. EcoRI expression caused a shift of the infection profile towards higher MOIs enabling cell survival up to an MOI of 0.3 in the presence of ≥2.5 µM daunorubicin, and resulting in a less pronounced cell lysis at higher MOIs. EcoRV and EcoP1_I showed comparable infection dynamics, with cell survival observed at MOIs < 0.3 under all daunorubicin conditions, which was slightly more pronounced for EcoRV. Remarkably, the Abi-like phenotype of the control and during EcoRI expression at MOIs of 1.25 was suppressed at daunorubicin concentrations of <2.5 µM.

Since members of the *Markadamsvirinae* subfamily appear to be more resistant to defense via RM systems, we also analyzed synergistic interactions of daunorubicin and RM systems during infection with the daunorubicin-sensitive *Changchunvirus* Bas09 *(Drexlerviridae)* which shows high sensitivity to type II and III RM systems but resistance to the type I RM system EcoCFT_I. As expected due to the absence of multiple recognition sites, expression of EcoCFT_I exhibited the same phenotype as the empty plasmid control showing the Abi phenotype at high MOIs and 2.5 µM daunorubicin. An intermediate effect was observed for the type III RM system EcoCFT_II, leading to a delayed culture collapse at a low MOI in the absence and no more growth defect in the presence of daunorubicin. The most pronounced effect was observed for EcoRI, which strongly retarded the phage-mediated cell lysis under normal infection conditions and completely prevented the culture collapse and Abi phenotype for both MOIs upon addition of daunorubicin (*SI Appendix*, Fig. S5*A*). Checking for additive or synergistic effects revealed a synergistic interaction of EcoCFT_II and EcoRI with daunorubicin for both or the higher applied MOIs, respectively (*SI Appendix*, Fig. S5*B*). Checkerboard-like assays with Bas09 and the chosen RM systems further supported the synergistic action (*SI Appendix*, Fig. S5*C*).

Taken together, daunorubicin and restriction-modification systems can synergistically protect cells from phage-induced cell lysis or death, thereby allowing survival of the entire population. These results emphasize the intricate interaction between different lines and highlight the context dependency of defense phenotypes.

## Discussion

Recent years have witnessed the discovery of numerous novel antiphage defense systems in bacteria, profoundly expanding our understanding of bacterial immunity. Beyond RNA- or protein-based systems also defense based on the activity of small antiphage molecules emerged as an underappreciated layer of phage defense (8, 9, 13, 28). Among the most prominent examples are DNA-intercalating anthracyclines produced and secreted by *Streptomyces*, which were previously shown to block infection of various dsDNA phages at an early step of the phage life cycle (9). In this study, we used the *E. coli* BASEL phage collection (17) to dissect the mechanism of action of DNA-intercalating antiphage molecules, focusing specifically on the anthracycline daunorubicin. While earlier work demonstrated a broad inhibitory effect, our systematic screening revealed pronounced phage-specific differences in phage sensitivity, with members of the *Drexlerviridae* (Bas1-13), *Demerecviridae* (Bas26-34), and *Vequintaviruses* (Bas48-59) showing particularly high sensitivity to DNA-intercalating antiphage compounds. Using the immunocompromised *E. coli* K-12 MG1655 ΔRM strain (17), we observed an “abortive infection” (Abi)-like phenotype for daunorubicin-sensitive phages (6, 7). This was not only evidenced by applying high MOIs leading to the typical “Abi-like” culture collapse or growth stagnation, but was also shown via live cell imaging in microfluidic chip devices, confirming growth arrest followed by membrane permeabilization of infected cells in the presence of daunorubicin (Fig. 2).

In our screening, members of the *Tevenvirinae*, including the model phage T4, exhibited a remarkably high resistance to different DNA-intercalating antiphage molecules (Fig. 2*F*). Although phage epigenomic modifications are well known to protect phages from diverse nucleic acid–targeting defense systems (18), our initial hypothesis, that the enhanced daunorubicin resistance of the *Tequatrovirus* T4 might result from DNA hypermodification via hydroxymethyl glycosylation, was disproved.

Using the *Tequintavirus* Bas33, we observed that in the presence of daunorubicin transcription and translation were confined to the pre-early phage gene region injected during the FST (23). Furthermore, no phage DNA replication could be detected under daunorubicin pressure. Accordingly, we hypothesize that daunorubicin blocks Bas33 infection at FST level, either by preventing progression of transcription and replication of injected phage DNA regions (e.g., via DNA intercalation) or by directly preventing SST. In the latter case, only the first ~9% of the Bas33 genome would be delivered into the host cell. However, the current methodology does not allow us to distinguish between these possibilities, and it remains possible that both mechanisms contribute.

The molecular mechanism underlying SST still remains poorly understood. The injection stop signal (ISS) has been proposed to reside at the phage-host interface and is characterized by multiple repeats, inverted repeats, palindromic motifs, and DnaA boxes. These features are thought to facilitate the formation of secondary structures and/or interaction with host components physically blocking SST (23, 24, 29). The phage proteins A1 and A2, which are essential for SST, may involve DNA releasing the blockage, which in turn might be blocked by the DNA intercalating activity of daunorubicin. The second step transfer typically results in the repression of pre-early phage genes due to a shift in RNA polymerase specificity to early phage gene expression upon association with A1, which was obviously absent under daunorubicin pressure. The deoxyribonucleotide 5′-monophosphatase (Dmp) promotes infection by further degrading host nucleotides during takeover (23). Additional toxicity to the host arises from the host division inhibitor (Hdi), which disrupts FtsZ ring formation, and the Ung-dependent nickase, which exploits Ung to target and cleave dUMP-containing DNA (30, 31). Hence, the continued expression of the pre-early host takeover genes is likely to have toxic effects on the host cell as indicated by the continuous DNA degradation under daunorubicin pressure for tequintaviruses, leading to cell death of the infected cell.

Similar to the pre-early proteins A1 and A2 of phage T5 and Bas33, many early phage proteins harm their host as an intrinsic part of the infection process (23, 32, 33). Accordingly, delaying or inhibiting the shut-off of these host takeover mechanisms, and the consequent accumulation of (pre-)early phage proteins, likely underlies the observed “Abi”-like phenotype. Importantly, in this case, cell death is not a result of a defense mechanism directed against host components, but rather an indirect effect from targeting phage components - defined as mutual destruction (7). A similar phenotype was observed for CRISPR-Cas targeting the pre-early genes of T5, which also enforced abortive infection (25). The distinction between classical Abi and mutual destruction caused by toxic phage products underscores the critical need to disentangle mechanistic cause from phenotypic outcome when interpreting abortive infection-like responses.

We furthermore show that the respective defense outcome is context-dependent. While we do observe cell death in an immunocompromised strain background, the synergistic interaction of daunorubicin and nucleic-acid targeting defenses (RM systems EcoRV and EcoP1, Fig. 4) counteracted the Abi phenotype and enabled survival of the infected cells. This effect was dependent on the respective recognition sites within the pre-early phage gene region. Under these conditions, an assumed combined action of inhibiting infection progression by daunorubicin and restricting of FST regions by RM systems is likely to prevent the accumulation of toxic phage products, thereby enabling cellular recovery. These findings align with recent studies emphasizing the context dependency of phage defense mechanisms (27). Another example involves type VI CRISPR systems, in which Cas13-mediated cleavage of mRNA targets would typically induce dormancy. However, in strains additionally carrying a RM system, initial dormancy is followed by recovery and resumed cellular growth (34). This context dependency even extends to the action of antibiotics as shown for the bactericidal activity of antifolate antibiotics in the presence of the CBASS antiphage defense system in **Vibrio* cholera* (35). Taken together, these studies highlight the complex interplay between small molecules and the different layers of the bacterial immune system, underscoring how the genetic makeup of the host strain shapes the resulting defense phenotype.

## Materials and Methods

### Bacterial Strains and Infection Conditions.

All bacterial strains, phages, and plasmids used in this study are listed in *SI Appendix*, Tables S1–S3, respectively. During this study, we observed that our variant of Bas33 carries a deletion (Bas33 Δ69,093 to 78,836 bp; *SI Appendix*, Fig. S6) at a locus where similar deletions had also been previously observed for variants of its close relative T5 (36). Since phenotypes observed with Bas33 were consistent with those for other *Markadamsvirinae* phages (Figs. 1 and 2) this deletion does not affect the phenotypes described in this manuscript.

*E. coli* cultures were inoculated from single colonies grown on LB agar plates in 5 mL LB medium supplemented with the indicated antibiotics and cultivated for 16 h at 37 °C and 170 rpm. For infection assays in liquid cultures, 300 µL main cultures were inoculated from precultures in the same medium to the desired OD_600_ and cultivated in the GrowthProfiler microcultivation system (Enzyscreen, Heemstede, The Netherlands) at 225 rpm and 37 °C using a 10 min imaging interval. Daunorubicin (dissolved in DMSO) was added to the main cultures to the indicated concentration prior to phage infection and “Red values” were recorded to monitor growth.

For omics-approaches, cultivations were scaled up to shaking flasks and cultivation was done at 150 rpm and 37 °C. Daunorubicin was added directly after inoculation, after which the cells were incubated for 5 min before the phages were added at the indicated MOI. If not indicated otherwise, infection assays were done in three independent biological replicates.

For double-agar overlay assays, *E. coli* K-12 MG1655 ΔRM precultures were used to inoculate LB soft-agar [0.4% (w/v)] to an OD_600_ of 0.2 and poured on LB agar plates. Decimal dilution series of phages in SM buffer (50 mM Tris-HCl, 100 mM NaCl, 8 mM MgSO_4_, pH 7.5) were spotted on top. Daunorubicin was added to both agar layers to the indicated concentrations.

### Phage Amplification and Purification.

For phage amplification, 5 mL *E. coli* K-12 MG1655 ΔRM precultures in LB medium were used to inoculate 70 mL main cultures in the same medium to a starting OD_600_ of 0.2. Cells were cultivated at 37 °C and 120 rpm for 90 min before phages were added to an MOI of 0.01. Supernatant was harvested after culture clearance via centrifugation at 5,000 *g* and 4 °C for 10 min. After sterile filtration, the phage lysate was again centrifuged at 37,000 *g* and 4 °C for 90 min. The phage pellet was resuspended in 1 ml TM buffer (50 mM Tris-HCl, 10 mM MgCl_2_, pH 7.5) and stacked on top of a sucrose gradient [0 to 45% (w/v); in TM buffer]. Ultracentrifugation was performed at 70,000 *g* and 4 °C for 20 min. The phage band was extracted using a syringe with needle and transferred to a fresh ultracentrifugation tube. 4 mL SM buffer were added and centrifugation was repeated at 70,000 *g* and 4 °C for 90 min. The phage pellet was finally resuspended in 4 mL SM buffer, sterile filtrated, and titers were determined via double-agar overlay assays.

### Fluorescence Microscopy.

To visualize intracellular DNA via DAPI staining, phage infection was performed as described in “Bacterial strains and infection conditions” with the following deviations: Cultures with an initial OD_600_ of 0.5 were cultivated for 45 min at 37 °C and 120 rpm before phages were added at an MOI of 5. At the respective time points, 500 µL samples were collected and cell-phage interactions were fixed by adding a final concentration of 4% paraformaldehyde for 15 min at room temperature. Subsequently, cell suspensions were centrifuged and resuspended in 200 µL PBS. For DNA staining, DAPI was added to a final concentration of 5 µg/mL. After incubating for 10 min at room temperature in the dark, the cells were washed once and resuspended in 50 µL of PBS. Fluorescence microscopic imaging was executed with the Axio Imager M2 phase contrast microscope (Zeiss, Germany). Therefore, 2 µL cells were fixed on 2% agarose slides and imaged with a Plan-Apochromat 100×, 1.40 Oil phase contrast oil-immersion objective and the filter set (Zeiss 49) for DAPI fluorescence. The pictures were taken using the AxioVision 4.8.2 software (Zeiss).

### Live Cell Imaging in Microfluidic Devices.

Single-cell analysis of infection dynamics was performed using an in-house developed microfluidic platform (37, 38). Cultivation of *E. coli* K-12 MG1655 ΔRM was performed in 60 × 60 µM chambers (height: 1,020 to 1,040 nM) flushed with LB medium containing 20 µM propidium iodide and 2.5 µM daunorubicin if indicated. A concentration of 10^9^ PFU/mL phages was continuously added with the medium flow at a flow rate of 200 nL/min. Growth and cell fate was recorded in 5 min intervals using phase contrast and the optical filter TexasRed (excitation, 560/40 nm; dichroic, 595; emission, 630/60 nm) at an exposure time of 200 ms and 100 ms, respectively. Preparation of image cutouts and adjustments of lookup tables (LUTs) were performed using NIS-Elements BR 5.30.03 (64 bit) and ImageJ 1.54f (39). Single-cell analysis has been performed based on acia-workflows (40) with custom single-cell analysis. The amount of lysed cells was determined by 1) segmenting cells using Omnipose (41), 2) cell size based filtering, and 3) computing the temporal cell population development (based on count and area) averaged over triplicates. The 50% drop from the global maximum quantifies the cell lysing progress. To determine single-cell PI fluorescence readouts, cell segments were tracked using trackastra (42) and the fluorescence development for every individual cell was measured.

### Quantification of Daunorubicin Uptake.

For measurement of daunorubicin uptake upon phage infection, *E. coli* K-12 MG1655 ΔRM was cultivated as described in “Bacterial strains and infection conditions” using an initial OD_600_ of 1.0 and an MOI of 2.5. Cultivation was performed in 1 mL Eppendorf tubes at 37 °C and 500 rpm. At the indicated time points, 250 µL cells were harvested by centrifugation at 16,000 *g* and 4 °C for 2 min and subsequently resuspended in 250 µL PBS. For measurement, 2 µL cells were diluted in 1 mL dd H_2_O and daunorubicin uptake was quantified via fluorescence measurements with the Cytek® Aurora flow cytometer using the detectors of the 488 nm laser. The gate was set up to gate on bacteria in FSC/SSC, followed by doublet exclusion by FSC-A/FSC-H. A gate for positive events was set based on unstained controls capturing 0.1% of the population at linear gate. Readouts were done for the percentage positive cells as well as for the MFI (mean of fluorescence) of the entire singlet population to monitor the shift in signal intensities.

### Long-Read Sequencing of Phage–Bacteria Complexes.

*E. coli* K-12 MG1655 ΔRM was cultivated as described in Bacterial strains and infection conditions using an initial OD_600_ of 0.8 and MOI of 2.5 in a total volume of 10 mL LB medium ± 5 µM daunorubicin. After 20 min of infection, cultures were harvested by centrifugation at 5,000 *g* and 4 °C for 10 min and washed two times with 10 mL 0.9% (w/v) NaCl to remove unbound phages. For DNA isolation, the cells were resuspended in 1 mL Tris buffer (20 mM Tris-HCl, 300 mM NaCl, pH 8.0), transferred to cell disruption tubes containing glass beads, and disrupted twice at 6,500 *g* for 20 s using a PreCellys® cell disrupter. After centrifugation at 16,000 *g* and 4 °C for 5 min, supernatants were transferred to a fresh tubes and RNA and protein digestion was performed with 200 µg/mL RNase A for 30 min at 30 °C and 300 µg/mL proteinase K for 2 h at 55 °C, respectively. Finally, the bacterial and phage DNA was purified using three cycles of phenol/chloroform/isoamylalcohol extraction and three cycles of chloroform extraction. The DNA was then precipitated using 0.1 volumes of 3 M sodium acetate (CH_3_COONa) and 2.5 volumes of absolute ethanol at −20 °C for 16 h, dried, and resuspended in 50 µL dd.H_2_O. Total DNA was subjected to Oxford Nanopore sequencing using the Native Barcoding Kit (SQK-NBD114.24) on an R10.4.1 flow cell (FLO-PRO114M) following the manufacturer’s instructions (Oxford Nanopore Technologies, UK). Details of the sequencing analysis including basecalling, demultiplexing, mapping, and filtering are provided in *SI Appendix*, *Text S1*.

### Transcriptomics via RNA-seq.

For transcriptomic analyses, cells were cultivated and infected as for “Long-read sequencing of phage–bacteria complexes” in a total volume of 25 mL LB medium.

At 20 min post infection, cells were harvested on ice at 5,000 *g* and 4 °C for 7 min and cell pellets were frozen in liquid nitrogen. Subsequent RNA isolation was done with the Monarch Total RNA Miniprep Kit (NEB, MA, U.S.) and concentrations were determined using a NanoDrop Spectrophotometer. Depletion of rRNA, library preparation, and paired-end sequencing on an Illumina NovaSeq platform with approximately 10 million read pairs per sample was performed at GENEWIZ from Azenta Life Sciences (Leipzig, Germany). RNA-seq results were analyzed using the CLC genomics workbench v.20 (Qiagen, Germany) as described in Wiechert et al. (43). Briefly, after pairing of reads, quality controls were conducted and raw reads were trimmed (chosen settings: quality trim; quality limit: 0.05; ambiguous trim: yes; ambiguous limit: 2; Remove 5’ and 3’ terminal nucleotides: no; Maximum length: 150; Trim from side: 3’ end; discard short or long reads: no). Subsequently, the “RNA-Seq Analysis” tool of was used to calculate the transcripts per million (TPM) (chosen parameters: reference sequence: combined genomes of Bas33_Jül_ (GCA_982375495) and *E. coli* K-12 MG1655 (NCBI:NC_000913.3); use spike-in controls: no; mismatch cost 2; insertion cost 3; deletion cost 3; length fraction 0.9; similarity fraction 0.9; maximum number of hits for a read: 10; strand specificity: both; library type: bulk). The “Differential Expression for RNA-seq” tool was applied for identifying significantly differentially expressed genes using a stringent threshold (FDR-*p*-values ≤ 0.01 and |log2 FC| ≥ 2). Differential expression statistics were derived from a generalized linear model fitted under a negative binomial distribution. To account for multiple hypothesis testing, *p*-values were adjusted using the Benjamini–Hochberg method controlling the false discovery rate (FDR) (44).

### Transcriptomics via RT-qPCR.

For quantification of single gene transcription of the Bas33 phage, cultivation, cell harvesting, and RNA isolation were performed at the indicated time points as described above. RT-qPCR was conducted with the Luna One-Step RT-qPCR Kit (New England Biolabs, Ipswich, MA) according to the manufacturer’s instructions in the qTower (Analytik Jena, Jena) using an input amount of 25 ng total RNA. Transcripts of *bas33_0180* (coding for A2 protein), *bas33_0182* (coding for A1 protein), and *bas33_0147* (coding for a homing endonuclease) were quantified using the *atpD* of *E. coli* K-12 MG1655 ΔRM as reference gene. Standard curves were included for all amplicons covering five log changes (PCR efficiency: 0.87 ≤ x ≤ 1.07; R^2^: 0.97 ≤ x ≤ 0.999, Slope: −3.69 ≤ x ≤ −3.11) and melting curves served as control for amplification specificity. Data were analyzed with qPCRsoft 3.1 (Analytik Jena, Jena, Germany) and relative concentrations were calculated according to the 2^−ΔCt^ method (45). Primers used for amplification are provided in *SI Appendix*, Table S4.

### Proteomics via LC-MS.

For proteome analysis, the biological triplicates were taken from the same cultivation batch as for the transcriptomic analyses via RNA-seq by harvesting 2 mL cells at 16,000 g and 4 °C for 2 min and subsequent freezing of cell pellets in liquid nitrogen. A detailed description of the sample preparation procedure for proteome measurements is provided in *SI Appendix*, *Text S1*.

### Declaration of Generative AI and AI-Assisted Technologies in the Writing Process.

During the preparation of this work, the authors used ChatGPT in order to improve language of the manuscript. After using this tool, the authors reviewed and edited the content as needed and take full responsibility for the content of the published article.

## Supplementary Material

Appendix 01 (PDF)

Dataset S01 (XLSX)

Movie S1. **Infection of *E. coli* K-12 MG1655 ΔRM with Bas33 in microfluidic chips using LB medium**. Microfluidic chambers were inoculated with *E. coli* cells and phages were added with the medium supply at a flow rate of 200 nl min-1. Propidium iodide was added to visualize membrane permeabilization.

Movie S2. **Infection of *E. coli* K-12 MG1655 ΔRM with Bas33 in microfluidic chips using LB medium with 2.5 μM daunorubicin**. Microfluidic chambers were inoculated with *E. coli* cells and phages were added with the medium supply at a flow rate of 200 nl min^-1^. Propidium iodide was added to visualize membrane permeabilization.

## Data Availability

The RNA-seq data were deposited in the European Nucleotide Archive (ENA) at EMBL-EBI (https://www.ebi.ac.uk/ena/browser/home) under accession number PRJEB101990. The long read sequencing data (control samples: B_Rx_libx; daunorubicin-treated samples: BD_Rx_libx) and the newly assembled Bas33_Jül_ genome (GCA_982375495) were deposited in the European Nucleotide Archive (ENA) at EMBL-EBI (https://www.ebi.ac.uk/ena/browser/home) under accession number PRJEB103991. The mass spectrometry proteomics data have been deposited to the ProteomeXchange Consortium via the PRIDE (46) partner repository with the dataset identifier PXD070949. All other data are included in the manuscript and/or supporting information.
