# Supplementary material for: DNA-intercalating antiphage molecules trigger abortive infection through mutual destruction and synergize with bacterial immunity
Source: Proc Natl Acad Sci U S A. 2026 Jun 3;123(23):e2602073123. doi: 10.1073/pnas.2602073123 (PMC13250531; doi:10.1073/pnas.2602073123)
Supplement: Supplementary file 1 — Appendix 01 (PDF) [file pnas.2602073123.sapp.pdf]

Supplemental information to:

DNA-intercalating antiphage molecules trigger abortive infection through 'mutual destruction' and synergize with bacterial immunity

Larissa Ernst[a,b], Cornelia Gätgens[a,b], Bente Rackow[a,b], Nadiia Pozhydaieva[c] , Elyès Gaaloul[c,h], Aileen Krüger[a,b], Johannes Seiffarth[a], Michelle Bund[a], Vivien Joisten-Rosenthal[d], Dietrich Kohlheyer[a], Björn Usadel[d,g],, Alexander Harms[e], Katharina Höfer[c,f,h], Julia Frunzke[a,b]

[a]Institute of Bio- und Geosciences, IBG-1: Biotechnology, Forschungszentrum Jülich, Jülich, Germany

[b] HHU Düsseldorf, Faculty of Mathematics and Natural Sciences Institute for Microbial Interactions, Heinrich-Heine-University Düsseldorf, Düsseldorf, Germany

[c]Max Planck Institute for Terrestrial Microbiology, Marburg, Germany

[d] HHU Düsseldorf, Faculty of Mathematics and Natural Sciences, Institute for Biological Data Science, Düsseldorf, Germany

[e]Institute of Food, Nutrition, and Health, Department of Health Sciences and Technology (D-HEST), ETH Zürich, Zürich, Switzerland

[f]Center for Synthetic Microbiology (SYNMIKRO), Philipps-Universität Marburg, Marburg, Germany

[g]Institute of Bio- and Geosciences, IBG-4: Bioinformatics, Forschungszentrum Jülich, Jülich, Germany

[h]Department of Pharmacy, Institute of Pharmaceutical Biology and Biotechnology, Philipps-Universität Marburg, Marburg, Germany.

Corresponding author: Julia Frunzke (j.frunzke@fz-juelich.de)

Supplementary Text

Text S1: Supplementary information on methods used in this study

Supplementary Dataset

Dataset S1: Transcripts per million (TPM) (A) and differential expression as log2 FC (B) upon Bas33 infection in presence and absence of 5 µM daunorubicin at 20 min post infection.

Supplementary Tables

Table S1: Bacterial strains used in this study

Table S2: Phages used in this study

Table S3: Plasmids used in this study

Table S4: Oligonucleotides used in this study

Supplementary Figures

Figure S1: DNA hypermodification showed no influence on sensitivity to daunorubicin

Figure S2: Growth and fluorescence analysis on single-cell level during cultivation of E. coli K-12 MG1655 ΔRM in microfluidic chips with applied propidium iodide (PI) stain

Figure S3: Influence of different DNA-intercalating agents on phage infection dynamics

Figure S4: Inhibition of the temperate phage λ by daunorubicin

Figure S5: DNA-targeting defense systems showed synergistic effects with daunorubicin towards Bas09 infection

Figure S6: Comparison of $\text{Bas33}_{\text{wildtype}}$ and $\text{Bas33}_{\text{Jülich}}$ genome

Supplementary Videos

Video S1: Infection of *E. coli* K-12 MG1655 ΔRM with Bas33 in microfluidic chips using LB medium.

Video S2: Infection of *E. coli* K-12 MG1655 ΔRM with Bas33 in microfluidic chips using LB medium with 2.5 µM daunorubicin.

Supplementary Text

Text S1: Supplementary information on methods used in this study

Analysis of long-read sequencing data

After Oxford Nanopore sequencing using the Native Barcoding Kit (SQK-NBD114.24) on an R10.4.1 flow cell (FLO-PRO114M) following the manufacturer's instructions (Oxford Nanopore Technologies, UK), raw sequencing data were basecalled with Dorado version 1.0.2 (Oxford Nanopore Technologies, UK) using the „super" accurate model dna_r10.4.1_e8.2_400bps_sup@v5.2.0 and the parameter --kit-name SQK-NBD114-24. Demultiplexing was performed with Dorado demux (version 1.0.2) using the --no-classify option. Reads from each replicate and condition were aligned against a combined reference consisting of E. coli K-12 MG1655 (NCBI:NC_000913.3) and Escherichia phage HildyBeyeler (Bas33; MZ501074.1, Jülich variant: GCA_982375495). Alignments were generated with minimap2 (version 2.30) using the presets -ax map-ont and the option --secondary=no (Li, 2018). Non-primary alignments, duplicates, and supplementary alignments were excluded with samtools view (version 1.20) using --excl-flags 3328 and converted to bam format with –bam (1). Reads mapping exclusively to the phage Bas33 reference were extracted with samtools fastq (version 1.20) using --excl-flags 3328 and converted to bam format with –bam. Reads mapping to the *Escherichia* phage HildyBeyeler (Bas33) reference were first extracted from the BAM file using samtools view with the -b option and the reference identifier MZ501074.1 to retain only reads aligned to the phage genome. The resulting BAM file was then converted to FASTQ format using samtools fastq (version 1.20) with the command samtools fastq -@{NUMBER_OF_THREADS} ${INPUT} > {OUTPUT} (1). Subsequent filtering using seqkit (version 2.9.0) retained only reads with a minimum quality score of 20 and a length of at least 10 kb (2). Long-read assembly of filtered reads was conducted with hifiasm (version 0.25.0) using the ont parameter (3). The resulting contig was then reorganized by identifying the terminase small subunit (*terS*) as the starting position and reorienting it to match the Bas33 reference genome. Annotation of the long read assembly was performed with Prodigal (version 2.6.3) to predict protein-coding sequences (4). Putative TerL and TerS proteins were identified by searching the UniProt database (UniProt Consortium 2017). Reviewed sequences were used to construct two BLAST protein databases with makeblastdb (version 2.15.0) using the options -

dbtype prot and -parse_seqids. Searches were then performed with blastp (version 2.15.0) using the parameters -evalue 1e-5, -max_target_seqs 10, and -outfmt 6 (5, 6). The identified *terS* hit was used to confirm that *terS* represents the true start position and that the contig orientation is consistent with the organization of the Bas33 reference genome. For comparative alignment, the re-assembled Bas33 genome from this study was combined with the E. coli K-12 MG1655 reference and re-analyzed using the same mapping and filtering workflow as described above. Only alignments to the assembled phage reference were retained and visualized with Integrative Genomics Viewer (IGV) (version 2.16.2) (7).

Phage abundance was normalized to host-mapped reads to account for differences in sequencing depth and sample composition. Specifically, phage reads were expressed as reads per million host-mapped reads (PPMH). Per-base coverage across the phage genome was calculated from the combined alignment BAM files using samtools depth with the -aa option to retain zero-coverage positions. The output was also restricted to the phage reference mappings using option -r. Phage coverage values were normalized by the number of host-mapped reads per sample and scaled to one million. For visualization, coverage was averaged across replicates and variability is shown with single replicates. Analyses were performed in R using standard packages (dplyr, ggplot2, readr, stringr).

Sample preparation and Liquid Chromatography-Mass Spectrometry (LC-MS) for peptide analysis

In order to prepare samples for proteome measurements, cells were thawed on ice and lysed by resuspending in 200 µL of lysis buffer (2% sodium lauroyl sarcosinate (SLS, w/v) in 100 mM ammonium bicarbonate (ABC) buffer), heating at 90°C for 15 min and 3 cycles of sonication (30s each) using a Hielscher Up200St ultrasonic processor (75% amplitude, 0.5 s / 0.5 s pulse). Subsequently, samples were centrifuged at 14,000 rpm for 5 min and supernatants were transferred into a new low protein binding tube. Protein concentrations were determined using a Pierce™ BCA Protein assay kit (ThermoFisher Scientific, 23250). Afterwards, 2 mM final tris(2-carboxyethyl)phosphine (TCEP, Tokyo Chemical Industry, ref. T1656) were added and samples were mixed and incubated at 90°C for 15 min to promote reduction of disulfide bridges. After cooling down, 4 mM iodoacetamide were added and samples were incubated in the dark for 30 min at room temperature. Afterwards, 30 µg of total lysate proteins were transferred to a new low protein binding

tube and sample volume was adjusted with lysis buffer to a total volume of 50 µL. Then, three volumes of 100 mM ABC buffer were added to the sample in order to dilute the final SLS concentration down to 0.5% (w/v). Then, SP3 magnetic beads slurry was prepared as follow: in a new tube, 20 µL of beads (Sera-Mag™ carboxylate-modified [E3] and [E7] magnetic beads, Cytiva) were mixed at a 1:1 ratio and washed twice with 300 µL of HPLC grade water. Then, beads were resuspended in 100 µL of water to produce readily usable slurry. Afterwards, 8 µL of bead slurry was added to the protein sample along with three sample volumes (600 µl) of 100% acetonitrile (ACN). Proteins were allowed to bind to the beads for 1 h at RT with 800 rpm shaking. Supernatant was discarded by using a magentic rack and beads were washed twice with 300 µl of 70% (v/v) ethanol, once with 300 µL of 100% (v/v) ACN before air drying for 5 min. Finally, protein digest was performed by adding 200 µL of 100 mM ABC buffer and 0.6 µg of sequencing-grade trypsin (Promega, V5111) overnight at 30°C with 1,000 rpm shaking. Supernatants were transferred to a new low protein binding tube and beads were resuspended in 100 µL of Millipore H_2O. After magnetic bead separation, supernatants were pooled with the previous ones and residual SLS was precipitated by addition of 4% (v/v) final concentration of trifluoroacetic acid (TFA, Serva 45641.01). Samples were centrifuged at 4°C at 14,000 rpm for 20 min and supernatants were purified with C18 columns (Chromabond, 730522.250). The solvent was evaporated with SpeedVac vacuum concentrations at 45°C and stored samples at -20°C before measurement.

Liquid Chromatography-Mass Spectrometry (LC-MS) for peptide analysis was carried out using a Vanquish Neo system coupled to an Orbitrap Exploris 480 mass spectrometer. Peptides were dissolved 0.1% trifluoroacetic acid and loaded onto a self-packed C18 (26 cm of 1.9 µm Reprosil-AQ, Dr. Maisch) column. The peptides were separated by an acetonitrile gradient running from 2 – 25 % solvent B (99.85% acetonitrile, 0.15% formic acid, v/v) over 45 min, followed by an additional increase of solvent B to 35% and 40 %, respectively, for 15 min at a flow rate of 300 nl/min. Solvent A composition was 0.15 % formic acid (v/v). Eluting peptides were analyzed in data independent acquisition (DIA) mode on the Exploris-MS. The funnel RF level was set to 40. Full MS resolution was set to 120.000 (m/z 200). Automatic gain control (AGC) target value for fragment spectra was set at 3000%. 45 windows of 14 Da were used with an overlap of 1 Da between m/z 320-950. Resolution was set to 15,000 and fill time to 22 ms. Stepped HCD collision energy of 25, 27.5, 30 % was used. MS1 data was acquired in profile, MS2 DIA data in centroid mode.

Analysis of DIA data was performed using the DIA-NN version 1.8 and 1.9 (8), respectively, using a uniprot protein database from *E. coli* with Bas33 phage proteins included (MZ501074; UniProt ID: 2852005) to generate a data set specific spectral library for the DIA analysis. The neural network based DIA-NN suite performed noise interference correction (mass correction, RT prediction and precursor/fragment co-elution correlation) and peptide precursor signal extraction of the DIA-NN raw data. The following parameters were used: Full tryptic digest was allowed with two missed cleavage sites, and oxidized methionines (variable) and carbamidomethylated cysteins (fixed). Match between runs and remove likely interferences were enabled. The precursor FDR was set to 1%. The neural network classifier was set to the single-pass mode. Quantification strategy was set to any LC ("high accuracy" for DIA-NN 1.8) and Quant UMS ("high precision" for DIA-NN 1.9). Cross-run normalization was set to RT-dependent. Library generation was set to smart profiling. DIA-NN outputs were further evaluated using the SafeQuant (9, 10) script modified to process DIA-NN outputs.

Data visualization was performed using GraphPad Prism 10.6.1. In brief, *E. coli* proteins that had a peptide count across replicates lower or equal to one count were filtered out and omitted from the analysis. Significance was calculated using a moderated t-test. The significance threshold for the $-\text{Log}_{10}$ (*p-value*) parameter was set to 1.3 and the threshold for the $\log_2$(ratio) value was set to 2. Phage proteins were considered reliably detected if their average peptide count across replicates was ≥ 2.

Supplementary Datasets

Dataset S1: Transcripts per million (TPM) (A) and differential expression as log2 FC (B) upon Bas33 infection in presence and absence of 5 µM daunorubicin at 20 min post infection. Analysis was carried out with CLC Workbench v.20 (Qiagen, Germany). A comprehensive table of all detected transcripts is provided, while a stringent threshold of |log2FC| ≥ 2 and FDR ≤ 0.01 was applied for analyses.

This table is provided as separate file: Dataset S1_Expression Browser_TPM_DifExp_Bas33_20 min

Supplementary Tables

Table S1: Bacterial strains used in this study

Bacterial strains	Description	Reference
***Escherichia coli* BW25113**	*F- Δ(araD-araB)567 ΔlacZ4787(::rrnB-3) λ- rph-1 Δ(rhaD-rhaB)568 hsdR514*	Keio Collection
***Escherichia coli* K-12 MG1655**	F^-, λ^-, *ilvG*$^-$, *rfb-50 rph-1*	
***Escherichia coli* K-12 MG1655 ΔRM**	*E. coli* K-12 MG1655 *Δmrr-hsdRMS-mcrBC ΔmcrA*	(11)
Escherichia coli* K-12 MG1655 ΔRM_pBR322_ΔP*tet	*E. coli* K-12 MG1655 ΔRM carrying the plasmid pBR322_ΔP_{tet}, Amp^R	This work
***Escherichia coli* K-12 MG1655 ΔRM_pEcoRI**	*E. coli* K-12 MG1655 ΔRM carrying the plasmid pEcoRI, Amp^R	This work
***Escherichia coli* K-12 MG1655 ΔRM_pAH213_EcoCFT_I**	*E. coli* K-12 MG1655 ΔRM carrying the plasmid pAH213_EcoCFT_I, Amp^R	This work
***Escherichia coli* K-12 MG1655 ΔRM_pAH213_EcoCFT_II**	*E. coli* K-12 MG1655 ΔRM carrying the plasmid pAH213_EcoCFT_II, Amp^R	This work
***Escherichia coli* K-12 MG1655 ΔRM_pAH213_EcoP1_I**	*E. coli* K-12 MG1655 ΔRM carrying the plasmid pAH213_EcoP1_I, Amp^R	This work

Table S2: Phages used in this study

Phage	Phage family	Subfamily	Bacterial host strain	Reference
Bas01	*Drexlerviridae*	*Braunvirinae*	*E. coli* K-12 MG1655 ΔRM	(11)
Bas02	*Drexlerviridae*	*Braunvirinae*		
Bas03	*Drexlerviridae*	*Braunvirinae*		
Bas04	*Drexlerviridae*	*Tempevirinae*		
Bas05	*Drexlerviridae*	*Tempevirinae*		
Bas06	*Drexlerviridae*	*Tempevirinae*		
Bas07	*Drexlerviridae*	*Tempevirinae*		
Bas08	*Drexlerviridae*	*Tempevirinae*		
Bas09	*Drexlerviridae*	*Tempevirinae*		
Bas10	*Drexlerviridae*	*Tempevirinae*		
Bas11	*Drexlerviridae*	*Tempevirinae*		
Bas12	*Drexlerviridae*	*Tunavirinae*		
Bas13	*Drexlerviridae*	*Tunavirinae*		
Bas14				
Bas15				
Bas16				
Bas17				
Bas18				
Bas19		*Queuovirinae*		
Bas20		*Queuovirinae*		
Bas21		*Queuovirinae*		
Bas22		*Queuovirinae*		
Bas23		*Queuovirinae*		
Bas24		*Queuovirinae*		
Bas25		*Queuovirinae*		
Bas26	*Demerecviridae*	*Markadamsvirinae*		
Bas27	*Demerecviridae*	*Markadamsvirinae*		
Bas28	*Demerecviridae*	*Markadamsvirinae*		
Bas29	*Demerecviridae*	*Markadamsvirinae*		
Bas30	*Demerecviridae*	*Markadamsvirinae*		
Bas31	*Demerecviridae*	*Markadamsvirinae*		
Bas32	*Demerecviridae*	*Markadamsvirinae*		
Bas33	*Demerecviridae*	*Markadamsvirinae*		
Bas34	*Demerecviridae*	*Markadamsvirinae*		
Bas35	*Straboviridae*	*Tevenvirinae*		
Bas36	*Straboviridae*	*Tevenvirinae*		
Bas37	*Straboviridae*	*Tevenvirinae*		
Bas38	*Straboviridae*	*Tevenvirinae*		
Bas39	*Straboviridae*	*Tevenvirinae*		
Bas40	*Straboviridae*	*Tevenvirinae*		
Bas41	*Straboviridae*	*Tevenvirinae*		
Bas42	*Straboviridae*	*Tevenvirinae*		
Bas43	*Straboviridae*	*Tevenvirinae*		
Bas44	*Straboviridae*	*Tevenvirinae*		
Bas45	*Straboviridae*	*Tevenvirinae*		

Bas46	*Straboviridae*	*Tevenvirinae*		
Bas47	*Straboviridae*	*Tevenvirinae*		
Bas48		*Vequintavirinae*		
Bas49		*Vequintavirinae*		
Bas50		*Vequintavirinae*		
Bas51		*Vequintavirinae*		
Bas52		*Vequintavirinae*		
Bas53		*Vequintavirinae*		
Bas54		*Vequintavirinae*		
Bas55		*Vequintavirinae*		
Bas56		*Vequintavirinae*		
Bas57		*Vequintavirinae*		
Bas58		*Vequintavirinae*		
Bas59		*Vequintavirinae*		
Bas60		*Stephanstirmvirinae*		
Bas61		*Stephanstirmvirinae*		
Bas62		*Stephanstirmvirinae*		
Bas63	*Autotranscriptaviridae*	*Ounavirinae*		
Bas64	*Autotranscriptaviridae*	*Studiervirinae*		
Bas65	*Autotranscriptaviridae*	*Studiervirinae*		
Bas66	*Autotranscriptaviridae*	*Studiervirinae*		
Bas67	*Autotranscriptaviridae*	*Studiervirinae*		
Bas68	*Autotranscriptaviridae*	*Studiervirinae*		
Bas69	*Schitoviridae*	*Enquatrovirinae*		
$Bas33_{Jülich}$ (Bas33 ∆69,093 - 78,836 bp)	*Demerecviridae*	*Markadamsvirinae*	*E. coli* K-12 MG1655 ∆RM	This study*
T4	*Straboviridae*	*Tevenvirinae*	*E. coli* B (DSM613)	DSM 4505
T5	*Demerecviridae*	*Markadamsvirinae*	*E. coli* B (DSM613)	DSM 16353
T7	*Autotranscriptaviridae*	*Studiervirinae*	*E. coli* B (DSM613)	DSM 4623
Lambda (λ)	*Zimmerviridae*	*Jacobvirinae*	*E. coli* LE392 (DSM4230)	DSMZ
T4 Δα/β gt = T4 Δgt	*Straboviridae*	*Tevenvirinae*	*E. coli* B (DSM613)	gifted by Marianne De-Paepe

*For all assays, the phage variant $Bas33_{Jülich\ (Jül)}$ was used (Figure S5).

Table S3: Plasmids used in this study

Plasmid	Description	Reference
**pBR322_ΔP*tet***	Amp^R, Derivative of pBR322, tetracycline resistance cassette deleted, empty vector control	(12)
**pEcoRI**	Amp^R, derivative of pBR322 expressing *E. coli* type II RM system EcoRI	
**pEcoRV**	Amp^R, derivative of pBR322 expressing *E. coli* type II RM system EcoRV	
**pAH213_EcoCFT_I**	Amp^R, derivative of pBR322 expressing *E. coli* type I RM system EcoCFT_I of *E. coli* CFT073	(11)
**pAH213_EcoCFT_II**	Amp^R, derivative of pBR322 expressing *E. coli* type III RM system EcoCFT_II of *E. coli* CFT073	
**pAH213_EcoP1_I**	Amp^R, derivative of pBR322 expressing *E. coli* type III RM system EcoP1_I of *E. coli* phage P1	

Table S4: Oligonucleotides used in this study

Name	Oligonucleotide sequence (5'-3')
**Bas33_qPCR_0180_fw**	GGCTTCTCCCGTGTCCGTTC
**Bas33_qPCR_0180_rv**	CCAGATCATCGGCGTCCTTGA
**Bas33_qPCR_0182_fw**	GCGGCGAAAGTAGCAAAAGCC
**Bas33_qPCR_0182_rv**	TATTGTTCAAGCGCGGCCAAAAAG
**Bas33_qPCR_0147_fw**	TCCCGAACAACGTGAAGATCGC
**Bas33_qPCR_0147_rv**	CGTGGCAATCTACAGCTTCCCAA
***E. coli*_qPCR_*atpD*_fw**	ACGGAATTTCTCAGCCATGGTCAGAC
***E. coli*_qPCR_*atpD*_rv**	GTGTTTGCGGGCGTAGGTGAAC

Supplementary Figures

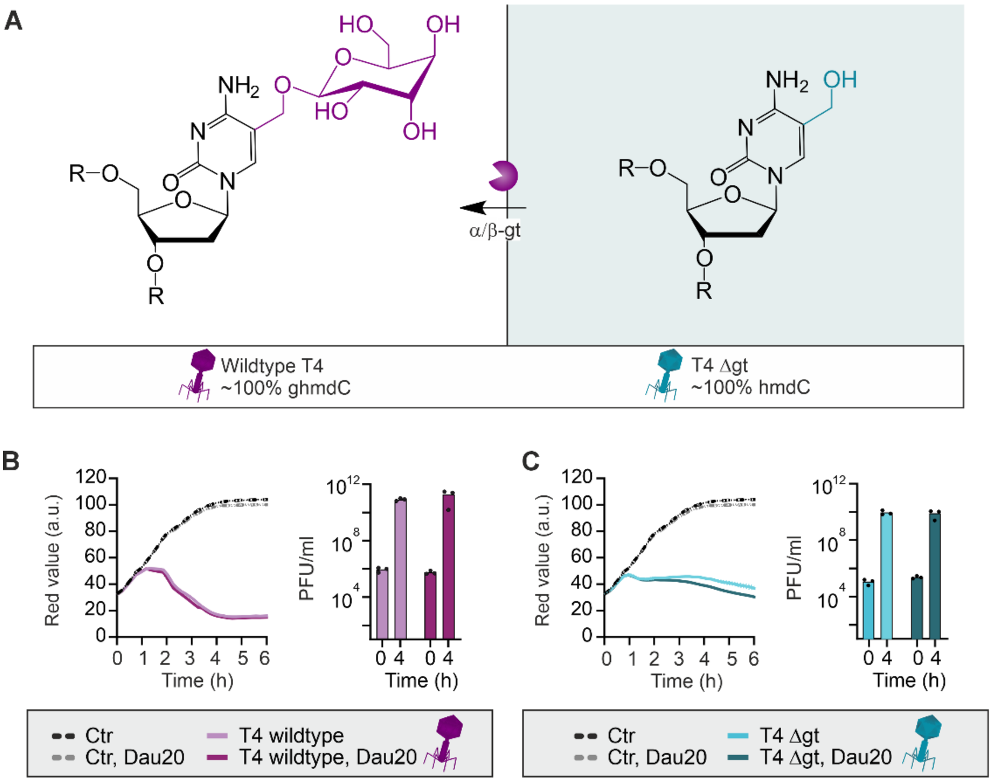

Figure S1: DNA hypermodification showed no influence on sensitivity to daunorubicin. A) Schematic representation of DNA modifications in wildtype T4 phages having glycosylated hydroxylmethyl-dCTPs (ghmdC) and T4 Δgt mutant phages lacking the glycosyl group (hmdC). B) Growth curves and titer development of *E. coli* K-12 MG1655 ΔRM upon infection with T4 phage in presence and absence of 20 µM daunorubicin. C) Growth curves and titer development of *E. coli* K-12 MG1655 ΔRM upon infection with T4 Δgt mutant phages in presence and absence of 20 µM daunorubicin (n=3, mean values with SD and single replicates are shown for growth and phage titers, respectively).

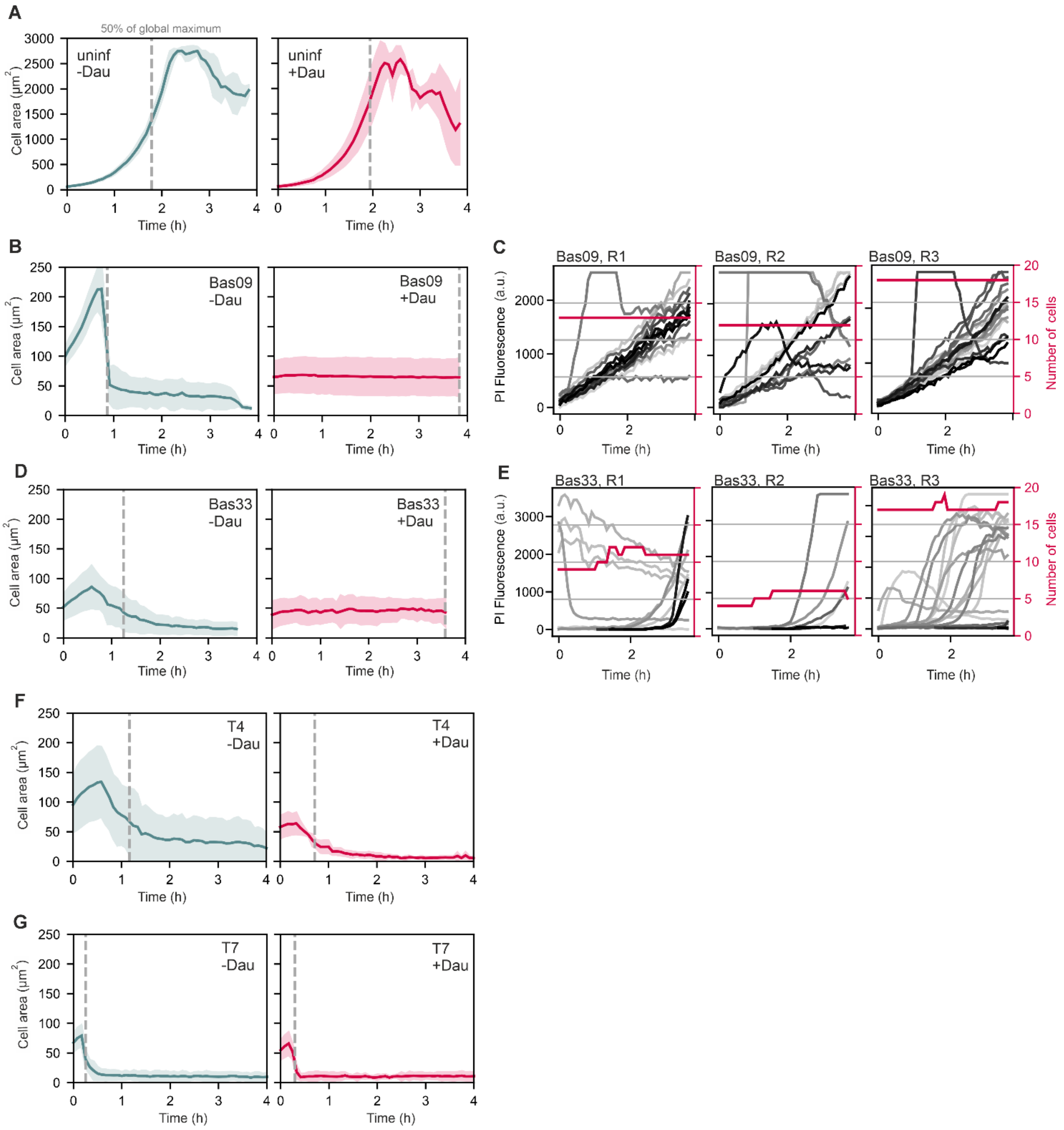

Figure S2: Growth and fluorescence analysis on single-cell level during cultivation of *E. coli* K-12 MG1655 ΔRM in microfluidic chips with applied propidium iodide (PI) stain. The dashed grey lines indicate the time points at which 50% of the global maximum cell area was reached. A) Cell area during cultivation of *E. coli* K-12 MG1655 ΔRM in presence and absence of 2.5 µM daunorubicin (n= 3, mean values with SD shown). B) Cell area during infection of *E. coli* K-12 MG1655 ΔRM with Bas09 in presence and absence of 2.5 µM daunorubicin (n= 3, mean values with SD shown). C) PI fluorescence per cell and number of cells upon infection of *E. coli* K-12 MG1655 ΔRM with Bas09 in presence of 2.5 µM daunorubicin, shown for the single replicates. D) Cell area during infection of *E. coli* K-12 MG1655 ΔRM with Bas33 in presence and absence of 2.5 µM daunorubicin (n= 3, mean values with SD shown). E) PI fluorescence per cell and number of cells upon infection of *E. coli* K-12 MG1655 ΔRM with Bas33 in presence of 2.5 µM daunorubicin, shown for the single replicates. F) Cell area during infection of *E. coli* K-12 MG1655 ΔRM with T4 in presence and absence of 2.5 µM daunorubicin (n= 3, mean values with SD shown). G) Cell area during infection of *E. coli* K-12 MG1655 ΔRM with T7 in presence and absence of 2.5 µM daunorubicin (n= 3, mean values with SD shown).

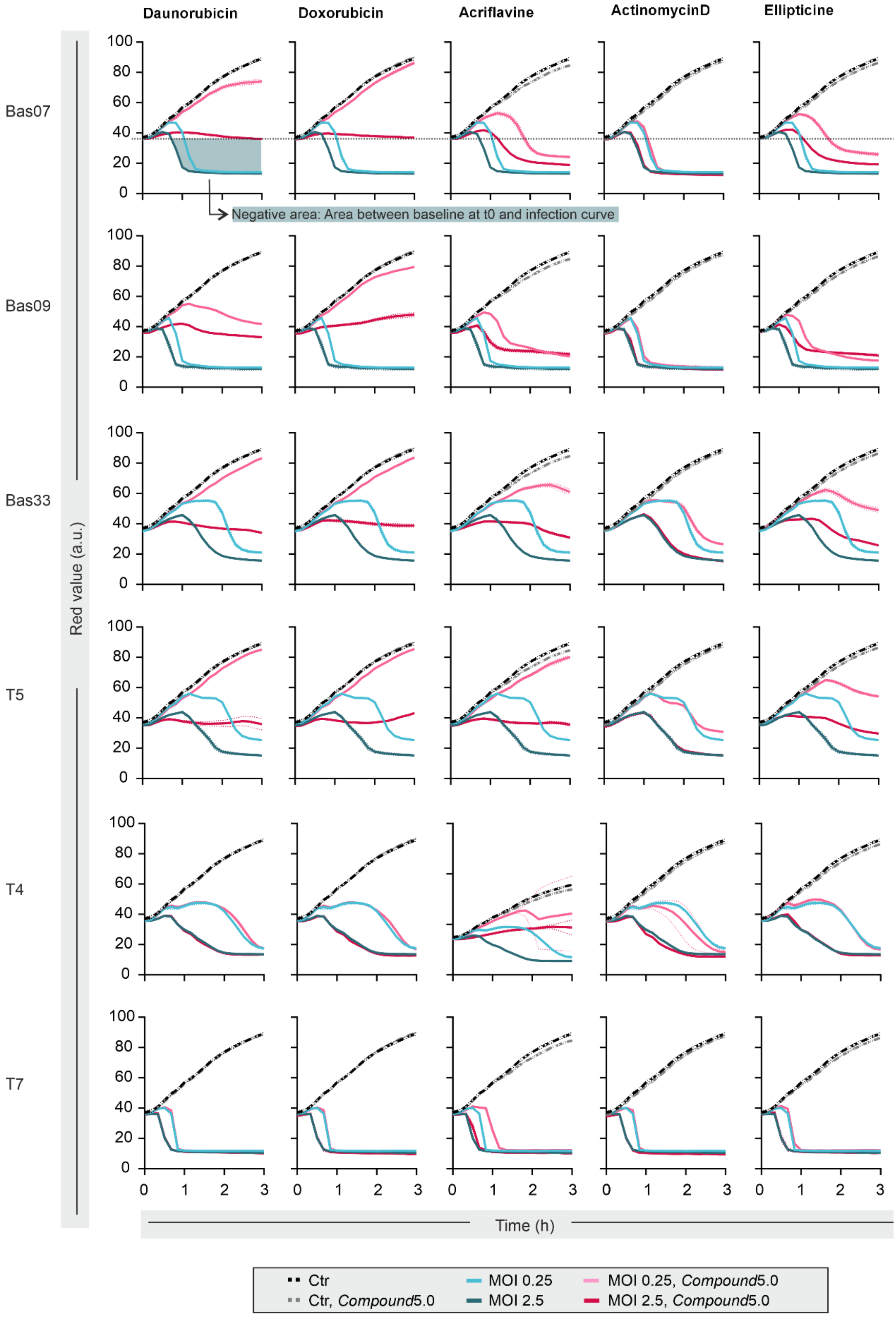

Figure S3: Influence of different DNA-intercalating agents on phage infection dynamics. Growth curves of *E. coli* K-12 MG1655 ΔRM upon infection with indicated phages in presence and absence of either 5 µM daunorubicin, doxorubicin, actinomycin D, ellipticine or 5 µg/ml acriflavine. For Bas07 and infection in presence of daunorubicin, principle of AUC calculation shown in Figure 2D is indicated by the dotted line showing the baseline (corresponds to 'Red value' at t_0) and the grey area showing the respective AUC (n= 3, mean values with SD shown).

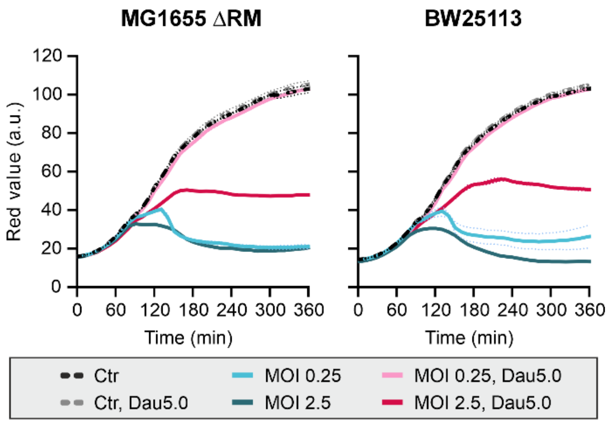

Figure S4: Inhibition of the temperate phage λ by daunorubicin. Growth curves of *E. coli* K-12 MG1655 ΔRM and BW25113 upon infection with phage λ in presence and absence of 5 µM daunorubicin (n= 3, mean values with SD shown).

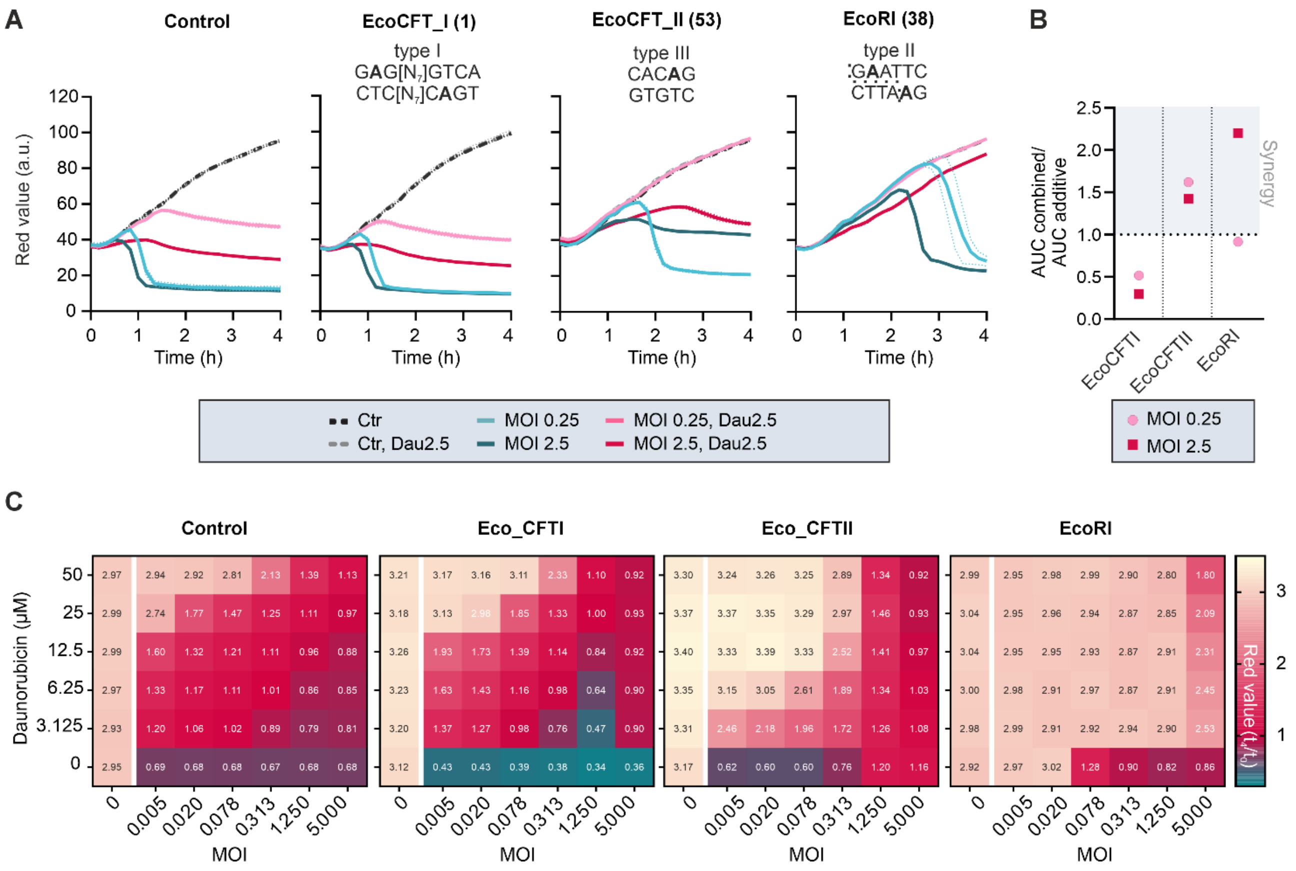

Figure S5: DNA-targeting defense systems showed synergistic effects with daunorubicin towards Bas09 infection. A) Liquid infection assays of the phage Bas09 infecting *E. coli* MG1655 ΔRM strains carrying different RM systems. Assays were performed in presence and absence of 2.5 µM daunorubicin at low and high MOIs of 0.25 and 2.5 (n= 3, mean values with SD shown). B) Calculation of synergy between daunorubicin and the respective RM systems based on the „Area under the curve (AUC)" according to Wu*, et al.* (13) using 'Red values' recorded at t_0 as baseline. C) Checkerboard-like assays combining different MOIs of the phage Bas09 and different daunorubicin concentrations in the respective *E. coli* strains with distinct RM antiphage defense backgrounds. The panel shows the FC in 'Red values' value after 4 h of cultivation and infection, with the calculated values indicated in the heatmaps.

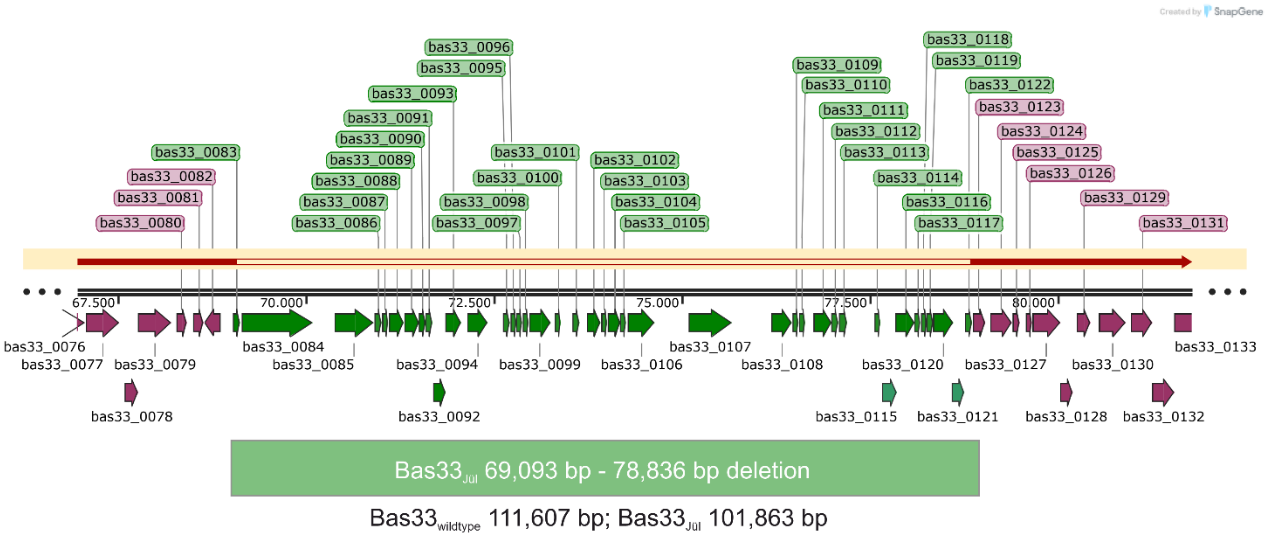

Figure S6: Comparison of Bas33$_{\text{wildtype}}$ and Bas33$_{\text{Jülich}}$ genome. The phage Bas33 variant used in all assays is characterized by a ~10 kbp deletion covering the basepairs 69,093 – 78,863. This region codes for several hypothetical proteins and tRNAs. A comparable deletion was already detected for T5$_{\text{Mos}}$ (14). However, the infection phenotype in the presence of daunorubicin was independently observed for several tequintaviruses (Figure 1). The genome alignment was created via SnapGene V8.1.1.

Supplementary Videos

Video S1: Infection of *E. coli* K-12 MG1655 ΔRM with Bas33 in microfluidic chips using LB medium. Microfluidic chambers were inoculated with *E. coli* cells and phages were added with the medium supply at a flow rate of 200 nl min^{-1}. Propidium iodide was added to visualize membrane permeabilization.

Video S2: Infection of *E. coli* K-12 MG1655 ΔRM with Bas33 in microfluidic chips using LB medium with 2.5 µM daunorubicin. Microfluidic chambers were inoculated with *E. coli* cells and phages were added with the medium supply at a flow rate of 200 nl min^{-1}. Propidium iodide was added to visualize membrane permeabilization.

The videos are provided as separate files:

- Video S1_Bas33 infection
- Video S2_Bas33 infection with 2.5 µM daunorubicin

References

1. P. Danecek *et al.*, Twelve years of SAMtools and BCFtools. *GigaScience* **10**, giab008 (2021).

2. W. Shen, B. Sipos, L. Zhao, SeqKit2: A Swiss army knife for sequence and alignment processing. *iMeta* **3**, e191 (2024).

3. H. Cheng, G. T. Concepcion, X. Feng, H. Zhang, H. Li, Haplotype-resolved de novo assembly using phased assembly graphs with hifiasm. *Nature Methods* **18**, 170-175 (2021).

4. D. Hyatt *et al.*, Prodigal: prokaryotic gene recognition and translation initiation site identification. *BMC Bioinformatics* **11**, 119 (2010).

5. S. F. Altschul, W. Gish, W. Miller, E. W. Myers, D. J. Lipman, Basic local alignment search tool. *Journal of Molecular Biology* **215**, 403-410 (1990).

6. C. Camacho *et al.*, BLAST+: architecture and applications. *BMC Bioinformatics* **10**, 421 (2009).

7. J. T. Robinson *et al.*, Integrative genomics viewer. *Nature Biotechnology* **29**, 24-26 (2011).

8. V. Demichev, C. B. Messner, S. I. Vernardis, K. S. Lilley, M. Ralser, DIA-NN: neural networks and interference correction enable deep proteome coverage in high throughput. *Nature Methods* **17**, 41-44 (2020).

9. E. Ahrné, L. Molzahn, T. Glatter, A. Schmidt, Critical assessment of proteome-wide label-free absolute abundance estimation strategies. *PROTEOMICS* **13**, 2567-2578 (2013).

10. T. Glatter *et al.*, Large-Scale Quantitative Assessment of Different In-Solution Protein Digestion Protocols Reveals Superior Cleavage Efficiency of Tandem Lys-C/Trypsin Proteolysis over Trypsin Digestion. *Journal of Proteome Research* **11**, 5145-5156 (2012).

11. E. Maffei *et al.*, Systematic exploration of *Escherichia coli* phage–host interactions with the BASEL phage collection. *PLOS Biology* **19**, e3001424 (2021).

12. M. Pleška *et al.*, Bacterial Autoimmunity Due to a Restriction-Modification System. *Current Biology* **26**, 404-409 (2016).

13. Y. Wu *et al.*, Bacterial defense systems exhibit synergistic anti-phage activity. *Cell Host & Microbe* **32**, 557-572.e556 (2024).

14. N. Burman *et al.*, A virally encoded tRNA neutralizes the PARIS antiviral defence system. *Nature* **634**, 424-431 (2024).
